# Stability Analysis of a Nonautonomous Diffusive Predator–Prey Model with Disease in the Prey and Beddington–DeAngelis Functional Response

**DOI:** 10.3390/biology14121779

**Published:** 2025-12-12

**Authors:** Yujie Zhang, Tao Jiang, Changyou Wang, Qi Shang

**Affiliations:** 1 School of Intelligent Medicine, Chengdu University of Tranditional Chinese Medicine, Chengdu 611137, China; zhangyujie@cdutcm.edu.cn; 2 Department of Basic Teaching, Dianchi College, Kunming 650228, China; 3 College of Applied Mathematics, Chengdu University of Information Technology, Chengdu 610225, China; shangqi1046@163.com

**Keywords:** eco-epidemic model, nonautonomous, periodic solution, persistence, global stability

## Abstract

Based on existing models, this paper incorporates some key ecological factors, thereby obtaining a class of eco-epidemiological models that can more objectively reflect natural phenomena. This model simultaneously integrates disease dynamics within the prey population and the Beddington–DeAngelis functional response, thus achieving an organic combination of ecological dynamics, epidemic transmission, and spatial movement under time-varying environmental conditions. The proposed framework significantly enhances ecological realism by simultaneously accounting for spatial dispersal, predator–prey interactions, disease transmission within prey species, and seasonal or temporal variations, providing a comprehensive mathematical tool for analyzing complex eco-epidemiological systems. The theoretical results obtained from this study can be summarized as follows: Firstly, the existence and uniqueness of globally positive solutions for any positive initial data are rigorously established, ensuring the well-posedness and biological feasibility of the model over extended temporal scales. Secondly, analytically tractable sufficient conditions for uniform population persistence are derived, which elucidate the mechanisms of species coexistence and biodiversity preservation even under sustained epidemiological pressure. Thirdly, by employing innovative applications of differential inequalities and fixed point theory, the existence and uniqueness of a positive spatially homogeneous periodic solution in the presence of time-periodic coefficients are conclusively demonstrated, capturing essential rhythmicities inherent in natural systems. Fourthly, through a sophisticated combination of the upper and lower solution method for parabolic partial differential equations and Lyapunov stability theory, the global asymptotic stability of this periodic solution is rigorously established, offering a powerful analytical guarantee for long-term predictive modeling. Beyond theoretical contributions, these research findings provide actionable insights and quantitative analytical tools to tackle pressing ecological and public health challenges. They facilitate the prediction of thresholds for maintaining ecosystem stability using real-world data, enable the analysis and assessment of disease persistence in spatially structured environments, and offer robust theoretical support for the planning and design of wildlife management and conservation strategies. The derived criteria support evidence-based decision-making in areas such as controlling zoonotic disease outbreaks, maintaining ecosystem stability, and mitigating anthropogenic impacts on ecological communities. A representative numerical case study has been integrated into the analysis to verify all of the theoretical findings. In doing so, it effectively highlights the model’s substantial theoretical value in informing policy-making and advancing sustainable ecosystem management practices.

## Simple Summary

This paper presents a new mathematical model that explores how predators, prey, and diseases interact in environments that change over time and space. The model combines several important ecological and epidemiological factors, including the movement of species across landscapes, the hunting behavior of predators (described by the Beddington–DeAngelis functional response), and the spread of diseases among prey populations. This comprehensive approach makes the model more realistic and useful for understanding complex natural systems. Key theoretical findings include proving that the model always produces biologically meaningful solutions for any set of initial conditions, ensuring its reliability over long periods. The study also identifies conditions under which species can coexist despite ongoing disease threats, helping to explain how biodiversity is maintained. Additionally, the model shows that, under certain time-varying conditions, there can be stable, repeating patterns in species populations, reflecting natural rhythms like seasons. The stability of these patterns is further confirmed, providing a solid basis for predicting long-term ecological outcomes. These theoretical insights offer practical tools for addressing real-world challenges, such as predicting when diseases might invade new areas, evaluating how diseases persist in different habitats, and designing strategies to manage wildlife and protect ecosystems. A numerical example is included to demonstrate the model’s accuracy and usefulness in real-world scenarios, highlighting its potential to guide policy and promote sustainable ecosystem management.

## 1. Introduction

The dynamic interplay between predators and their prey represents a fundamental topic in theoretical ecology due to its widespread occurrence and ecological importance [1,2]. Mathematical modeling serves as a crucial methodology for understanding the behavioral dynamics of predator–prey systems [3]. Central to this interaction is the functional response, which quantifies the per capita predation rate as a function of prey and predator densities [4]. While classical Holling-type responses consider only prey abundance, Arditi and Ginzburg (1989) introduced a ratio-dependent form of functional response [5], where predation rate depends on the ratio of prey to predators, thereby offering a more realistic representation of scenarios involving predator interference or competition. Subsequent research has further established the ecological relevance and mathematical richness of the ratio-dependent model. For instance, Conser et al. (1999) reinforced the conceptual basis of ratio-dependence through fundamental ecological principles [6], and numerous studies have since explored its dynamic implications across various contexts: Haque (2009) established stability conditions for autonomous systems [7]; Gao and Li (2013) identified Bogdanov–Takens bifurcation under a strong Allee effect [8]; Agrawal and Saleem (2015) demonstrated chaotic attractors in a three-species system [9]; Mandal (2018) incorporated stochasticity and Allee effects to study coexistence [10]; Jiang et al. (2020) employed comparison methods to analyze global stability in non-spatial systems [11]; Yu et al. (2023) investigated a ratio-dependent model with additional food supply, revealing complex dynamical patterns [12]; and Abbasi et al. (2025) recently studied an ecological model with Beddington–DeAngelis functional response and prey refuge, deriving different conditions that show how prey refuges impact the stability of various equilibrium points [13]. Notably, existing studies on predator–prey models with functional response have largely omitted the influence of spatial diffusion [14], which represents a significant gap in understanding how population dispersal and spatial heterogeneity affect these ecological interactions. Due to the inherent tendency of animal populations to exhibit involuntary aggregation behavior toward essential resources such as food and water, a novel model framework has been developed by incorporating diffusion terms into the above systems. This modified formulation provides a more accurate representation of the objective laws governing spatial population interactions. However, it is important to note that the conventional analytical approaches presented in the prior literature are not directly applicable to the systematic investigation of these revised models. The integration of diffusion mechanisms introduces additional complexity in parameter sensitivity analysis and characterization of asymptotic behavior, necessitating the development of specialized theoretical tools for comprehensive model evaluation.

In recent years, predator–prey model integrating diffusion mechanisms and functional responses have attracted growing interest in mathematical ecology due to their capacity to capture spatially explicit trophic interactions. These frameworks significantly enhance ecological realism by accounting for species movement and resource-driven aggregation. Ko and Ahn (2013) pioneered analysis of a diffusive system with one prey and two competing predators [15,16], establishing persistence criteria and global attractivity conditions. Building on this foundation, Yang et al. (2015) applied fixed point index theory to investigate a Leslie–Gower functional response model [17], deriving coexistence criteria. Wang (2017) later examined homogeneous Neumann boundary systems with Holling type-III responses [18], employing coincidence degree theory to demonstrate non-constant equilibria. Wu and Zhao (2020) incorporated Allee effects and threshold hunting [19], analyzing stability through Jacobian matrix methods. Recent advances include Yan and Zhang (2022) stabilizing Beddington–DeAngelis functional response systems [20], followed by Chen and Wu (2023) utilizing Leray–Schauder degree theory for spatiotemporal analysis [21]. Most recently, Chen et al. (2025) explored periodic solution stability in Beddington–DeAngelis systems [22]. However, most existing studies center on autonomous model. In reality, ecological parameters like birth and death rates, as well as interaction coefficients, naturally change over time due to seasonal cycles, environmental disruptions, and human impacts. As a result, nonautonomous reaction–diffusion models are more realistic for capturing complex time-varying dynamics. Yet, traditional methods based on eigenvalues are not adequate for analyzing the intricate behavior of these models. This discrepancy underscores the urgent need for new mathematical tools to systematically study the persistence, stability, and long-term dynamics of these ecologically vital nonautonomous systems.

The fields of theoretical ecology and epidemiology were historically studied in isolation, despite the numerous interconnections between them. Toward the end of the twentieth century, researchers began to develop integrated mathematical models in which both pathogens and predators act as natural enemies of a shared prey population. This led to the emergence of eco-epidemic model, which have since been extensively studied, revealing many complex ecological dynamics consistent with empirical observations [23,24,25]. In [26], the authors established a mathematical model to describe the interaction between a diseased fish population and its predators. Through analysis of the model, the authors proved the existence, uniqueness, and uniform boundedness of the solutions, as well as the stability of equilibrium points over a wide range of parameter values. A foundational contribution was made by Hethcote et al. [27], who proposed a three-compartment model in which a diseased prey population exhibits increased susceptibility to predation. Subsequent advances incorporated more realistic mechanisms such as nonlinear incidence rates; for instance, Capasso and Serio [28] introduced a saturated incidence term to account for behavioral changes and crowding effects during epidemics such as SARS. Nevertheless, most current eco-epidemiological model neglect two critical aspects: population dispersal and temporal variation in ecological parameters [29,30]. Many classical approaches, including those modeling disease in prey, predators, or both, assume homogeneous mixing and time-independent parameters, thereby ignoring the roles of spatial movement (e.g., diffusion) and seasonal or anthropogenic temporal drivers in shaping disease dynamics. For example, in 2021, Shaikh et al. [31] investigated the complex dynamics of the following eco-epidemic predator–prey system in which disease is transmitted among prey species.

$$\left\{\begin{array}{l} \frac{dx}{dt}=rx(1-\frac{x}{K})[r_{1}(t)-\frac{mxz}{A_{1}+x}-\beta xy],\\ \frac{dy}{dt}=\beta xy-\frac{myz}{A_{2}+y}-d_{1}y,\\ \frac{dz}{dt}=\mu +e_{1}\frac{mxz}{A_{1}+x}+e_{2}\frac{myz}{A_{2}+y}-d_{2}z. \end{array}\right.$$

In this system, x, y, and z denote the density of susceptible prey population, infected prey population, and predator population, respectively. $e_{1}$, $e_{2}$ are the conversion efficiencies due to consumption of susceptible and infected prey, respectively. $d_{1}$, $d_{2}$ are the death rates of susceptible prey and predator, respectively. μ is the growth rate of the predator due to additional food. β is the force of infection. m is the predator’s capture rate for both susceptible and infected prey. r is an intrinsic growth rate. K is the carrying capacity, and $A_{1}$ and $A_{2}$ are half-saturation constants. The authors have proved that the system possesses some important dynamical characteristics, including stable coexistence, Hopf bifurcation, period-doubling bifurcation, and chaotic behavior. In 2025, Majee et al. [32] studied the following reaction–diffusion eco-epidemic model:

$$\left\{\begin{array}{l} \frac{\partial S}{\partial t}-d_{1}\Delta S=rS(1-\frac{S+I}{K})-\lambda SI-\frac{\alpha_{1}(1-\rho )SP}{1+bP+c(1-\rho )S}-q_{1}ES,\\ \frac{\partial I}{\partial t}-d_{2}\Delta I=\lambda SI-\frac{mIP}{a+I}-\mu I-q_{2}EI,\\ \frac{\partial P}{\partial t}-d_{3}\Delta P=\frac{\alpha_{2}(1-\rho )SP}{1-bP+c(1-\rho )S}+\frac{\theta IP}{a+I}-dP-\delta P^{2}, \end{array}\right.$$

where S, I, and P denote the density of susceptible prey population, infected prey population, and predator population, respectively. In [32], the authors primarily focus on the interior equilibrium point, whose existence depends on the values of the system parameters. In the absence of diffusion, the system exhibits rich dynamical characteristics, such as Hopf bifurcation, chaotic phenomena, and so on. The authors analytically investigate the potential conditions for Turing instability within the context of diffusion. These simplifications stem largely from the analytical difficulties posed by combining diffusion, nonautonomous dynamics, and nonlinear functional responses, which together obstruct a full understanding of stability, persistence, and pattern formation [33,34]. Consequently, the existing model often fails to capture essential realistic features such as fear-induced dispersal [35], habitat fragmentation, or spatiotemporally heterogeneous control measures [36]. Addressing these limitations requires the development of new mathematical tools, including Lyapunov functions, fixed point theory, and analytical techniques for reaction–diffusion systems. Progress in this direction will not only advance theoretical biology but will also improve predictive model and support practical disease management in spatially structured and temporally varying environments, thereby contributing to the design of more effective public health and ecological policies.

Building upon the foregoing analysis and inspired by existing research, this paper investigates the global stability of a nonautonomous diffusive predator–prey model that incorporates disease dynamics within the prey population and a Beddington–DeAngelis functional response. The total population is divided into three compartments: susceptible prey, infected prey, and predators. Section 2 details the model formulation and underlying assumptions. Section 3 addresses the positivity and uniform persistence of solutions. The existence and stability of a positive spatially homogeneous periodic solution is established in Section 4. All key analytical findings are numerically validated using MATLAB in Section 5. Finally, Section 6 presents a general discussion of the results and their biological implications.

**Remark 1.** 
*The innovations and contributions of this study are summarized as follows: (1) A novel nonautonomous diffusive predator–prey model with disease in the prey population is proposed, incorporating Beddington–DeAngelis functional response, diffusion terms, and time-dependent coefficients. This formulation provides a more realistic representation of population interactions by simultaneously accounting for disease dynamics, spatial dispersal, and temporal environmental variability. (2) By integrating the comparison principle with fixed point theory, new analytical methods and theoretical frameworks are developed. These approaches establish the existence of a strictly positive spatially homogeneous periodic solution under a set of verifiable sufficient conditions, significantly simplifying the qualitative analysis of complex ecosystems. (3) Through the construction of a novel Lyapunov function and the application of upper and lower solution methods for parabolic partial differential equations, the global asymptotic stability of the positive periodic solution is rigorously demonstrated. The derived criteria provide explicit conditions for ensuring long-term ecological stability in spatially extended systems. (4) Compared to existing results [30,31,32,33,34], the findings presented in this study offer greater generality and analytical flexibility. The proposed framework enhances the applicability of Lotka–Volterra-type models to long-term ecological studies and offers practical insights for managing predator–prey systems subject to disease and spatial heterogeneity.*

## 2. Development of Mathematical Model

In this section, a nonautonomous reaction–diffusion model is developed to describe a spatially structured predator–prey system with disease dynamics in the prey population, with trophic interactions governed by the Beddington–DeAngelis functional response. In contrast to the conventional autonomous ordinary differential equation (ODE) model that assumes fixed parameters and spatial homogeneity, the proposed partial differential equation (PDE) framework incorporates explicit diffusion terms to represent spatial movement across heterogeneous landscapes, while its time-dependent coefficients allow the model to capture seasonal variations, resource fluctuations, and other temporal environmental drivers. This nonautonomous model integrates both local processes like disease transmission within the prey population (modeled through compartmental structures) and predation mechanisms involving predator interference and handling time, alongside spatial processes including population dispersal and habitat fragmentation effects. The formulation combines these elements within a unified framework that accounts for temporal environmental variability through nonautonomous parameters while capturing spatial dynamics via diffusion terms and spatially heterogeneous coefficients. By linking local ecological interactions with landscape-scale patterns, this approach enables analysis of how temporal fluctuations interact with spatial habitat structure to influence disease dynamics in predator–prey systems. By embracing spatiotemporal explicitness, the model offers a biologically more realistic and mechanistically enriched representation of ecosystem dynamics. It enables deeper insights into how spatial and temporal variability jointly influence disease propagation, trophic stability, and pattern formation, phenomena that classical autonomous ODE model are inherently unable to reveal. Thus, the present PDE approach significantly extends the theoretical foundation and practical applicability of eco-epidemiological modeling in non-equilibrium and heterogeneous environments.

Suppose there exists an ecological species that is susceptible to a lethal and contagious disease. When the disease is present, the total prey population is partitioned into two distinct groups. Let $X_{S}(x,t)$ and $X_{I}(x,t)$ represent the densities of the susceptible and infected populations, respectively, at location $x=(x_{1},\ldots ,x_{n})$ and time t. Furthermore, assume that another species preys on the diseased species, and its density at the same location and time is denoted by Y(x,t). In order to establish the eco-epidemiological model of this paper, we need the following assumptions:

(i) Only the prey population is affected by the infectious disease.
(ii) The susceptible prey population exhibits logistic growth with an intrinsic growth rate $r_{1}(t)$. Infected individuals die before reaching reproductive age and therefore do not contribute to population growth.
(iii) Disease transmission occurs vertically from susceptible to infected prey populations according to the law of mass action, characterized by the infection rate β(t).
(iv) Infected prey populations do not recover or regain health after infection.
(v) Disease transmission from infected prey to predators is precluded through any form of direct contact or feeding interaction.
(vi) The internal competition within the susceptible prey population stems from all prey populations, based on the fact that these prey populations utilize the same ecological resources. However, since infected prey have a weaker attack resistance against predators and thus predators only choose to prey on infected preys, the internal competition within the infected prey population is solely related to the infected population itself. This is because the higher the density of the infected prey population, the easier it is for predators to detect them, and consequently, the more likely they are to be preyed upon, leading to a faster decline in their population density.
(vii) The carrying capacity of the predator’s environment demonstrates a positive correlation with infected prey population density.
(viii) Predators exclusively prey upon infected individuals and do not consume susceptible prey. This assumption is ecologically justified by the reduced mobility and escape capabilities of infected prey compared to healthy individuals [30,37].
(ix) The functional response governing predator–infected prey interactions follows a Beddington–DeAngelis type formulation.
(x) The predator mortality rate is modeled as density-dependent with the term $d(t)Y^{2}(t)$, which captures either self-limitation within the predator population or increased mortality due to external predation. Self-limitation may arise from resource constraints other than food that become influential at high population densities. Alternatively, this term may reflect heightened predation pressure on the consumers themselves, as elevated consumer densities could attract more predators or increase individual vulnerability [38].

Based on the above assumptions, the eco-epidemiological model can be presented by the following set of partial differential equations:

(1) $$\left\{\begin{array}{l} \frac{\partial X_{S}(x,t)}{\partial t}-D_{1}(t)\Delta X_{S}(x,t)=X_{S}(x,t)\left[r_{1}(t)-b(t)(X_{S}(x,t)+X_{I}(x,t))-\beta (t)X_{I}(x,t)\right],\;(x,t)\in \Omega \times R^{+},\\ \frac{\partial X_{I}(x,t)}{\partial t}-D_{2}(t)\Delta X_{I}(x,t)=X_{I}(x,t)[-\delta (t)-\varepsilon (t)(X_{S}(x,t)+X_{I}(x,t))-c^{*}(t)X_{I}(x,t)+\beta (t)X_{S}(x,t)\\ \;\;\;\;\;\;\;\;\;\;\;\;\;-\frac{\alpha (t)Y(x,t)}{1+a(t)X_{I}(x,t)+e(t)Y(x,t)}],(x,t)\in \Omega \times R^{+},\\ \frac{\partial Y(x,t)}{\partial t}-D_{3}(t)\Delta Y(x,t)=Y(x,t)\left[-r_{2}(t)-d(t)Y(x,t)+\frac{\gamma (t)X_{I}(x,t)}{1+a(t)X_{I}(x,t)+e(t)Y(x,t)}\right],(x,t)\in \Omega \times R^{+}, \end{array}\right.$$

with the Neumann boundary and initial conditions

(2) $$\begin{array}{l} \;\;\;\frac{\partial X_{S}(x,t)}{\partial n}=\frac{\partial X_{I}(x,t)}{\partial n}=\frac{\partial Y(x,t)}{\partial n}=0,\;\;(x,t)\in \partial \Omega \times R^{+},\;\\ X_{S}(x,0)=\eta_{X0}(x,0)>0,\;X_{I}(x,0)=\eta_{I0}(x,0)>0,Y(x,0)=\eta_{Y0}(x,0)>0,x\in \Omega , \end{array}$$

where Ω is a bounded smooth domain in $R^{n}$ with boundary ∂Ω, Δ is a Laplace operator on Ω, and ∂/∂n represents the outward normal derivation on ∂Ω. All coefficients in the model are positive continuous and bounded functions defined on [0, +∞). From Table 1, one can discern the biological meaning associated with the other parameters in model (1).

According to assumptions (ii) and (iv), the disease that infected prey contract is an incurable and fatal one, and infected prey die before reaching adulthood meaning they have a relatively short survival time. As a result, the intraspecific competition among infected prey due to food is very weak. In other words, the parameter ε(t) is much smaller than parameters $c^{*}(t)$ and β(t). Let $c(t)=\varepsilon (t)+c^{*}(t),\;\psi (t)=\beta (t)-\varepsilon (t)$, then model (1) can be simplified into the following model:

(3) $$\left\{\begin{array}{l} \frac{\partial X_{S}(x,t)}{\partial t}-D_{1}(t)\Delta X_{S}(x,t)=X_{S}(x,t)\left[r_{1}(t)-b(t)(X_{S}(x,t)+X_{I}(x,t))-\beta (t)X_{I}(x,t)\right],\;(x,t)\in \Omega \times R^{+},\\ \frac{\partial X_{I}(x,t)}{\partial t}-D_{2}(t)\Delta X_{I}(x,t)=X_{I}(x,t)[-\delta (t)-c(t)X_{I}(x,t)+\psi (t)X_{S}(x,t)-\frac{\alpha (t)Y(x,t)}{1+a(t)X_{I}(x,t)+e(t)Y(x,t)}],(x,t)\in \Omega \times R^{+},\\ \frac{\partial Y(x,t)}{\partial t}-D_{3}(t)\Delta Y(x,t)=Y(x,t)\left[-r_{2}(t)-d(t)Y(x,t)+\frac{\gamma (t)X_{I}(x,t)}{1+a(t)X_{I}(x,t)+e(t)Y(x,t)}\right],(x,t)\in \Omega \times R^{+}, \end{array}\right.$$

**Remark 2.** 
*Model (2)–(3) established in this paper is new and has not been found to be studied by other scholars. It is particularly important to emphasize here that the model can be used to describe many real ecosystems in nature rather than mathematical models without an actual background. For example, in freshwater ecosystems, certain fish species such as carp and crucian carp may be infected with parasites like liver flukes and fingerworms. These parasites circulate exclusively within fish populations and do not infect predators such as herons or cormorants. However, predators will preferentially prey on infected fish because they may exhibit abnormal behaviors (such as slow swimming and slow response), making them more vulnerable to predation. Through the study of this model, the related population in a certain water area can be monitored or controlled artificially to maintain its ecological balance and stability.*

## 3. The Positivity of Solutions and Persistence of Model

This section presents some preliminary results, the persistence of the solution, and the definition of the upper and lower solutions; the definitions of a spatially homogeneous periodic solution and its global asymptotic stability can be found in reference [39].

**Definition 1.** 
*Suppose that* $\tilde{U}(x,t)\equiv ({\tilde{X}}_{S}(x,t),{\tilde{X}}_{I}(x,t),\tilde{Y}(x,t))$*,* $\hat{U}(x,t)=({\hat{X}}_{S}(x,t),{\hat{X}}_{I}(x,t),\hat{Y}(x,t))$ *, if* $\tilde{U}(x,t)\geq \hat{U}(x,t)$ *and for* $(x,t)\in \Omega \times R^{+}$

$$\begin{array}{l} \frac{\partial {\tilde{X}}_{S}(x,t)}{\partial t}-D_{1}(t)\Delta {\tilde{X}}_{S}(x,t)\geq {\tilde{X}}_{S}(x,t)[r_{1}(t)-b(t)({\tilde{X}}_{S}(x,t)+{\hat{X}}_{I}(x,t))-\beta (t){\hat{X}}_{I}(x,t)],\\ \frac{\partial {\tilde{X}}_{I}(x,t)}{\partial t}-D_{2}(t)\Delta {\tilde{X}}_{I}(x,t)\geq {\tilde{X}}_{I}(x,t)\left[-\delta (t)-c(t){\tilde{X}}_{I}(x,t)+\psi (t){\tilde{X}}_{S}(x,t)-\frac{\alpha (t)\hat{Y}(x,t)}{1+a(t){\tilde{X}}_{I}(x,t)+e(t)\hat{Y}(x,t)}\right],\\ \frac{\partial \tilde{Y}(x,t)}{\partial t}-D_{3}(t)\Delta \tilde{Y}(x,t)\geq \tilde{Y}(x,t)\left[-r_{2}(t)-d(t)\tilde{Y}(x,t)+\frac{\gamma (t){\tilde{X}}_{I}(x,t)}{1+a(t){\tilde{X}}_{I}(x,t)+\tilde{Y}(x,t)}\right],\\ \frac{\partial {\hat{X}}_{S}(x,t)}{\partial t}-D_{1}(t)\Delta {\hat{X}}_{S}(x,t)\leq {\hat{X}}_{S}(x,t)[r_{1}(t)-b(t)({\hat{X}}_{S}(x,t)+{\tilde{X}}_{I}(x,t))-\beta (t){\tilde{X}}_{I}(x,t)],\\ \frac{\partial {\hat{X}}_{I}(x,t)}{\partial t}-D_{2}(t)\Delta {\hat{X}}_{I}(x,t)\leq {\hat{X}}_{I}(x,t)\left[-\delta (t)-c(t){\hat{X}}_{I}(x,t)+\psi (t){\hat{X}}_{S}(x,t)-\frac{\alpha (t)\tilde{Y}(x,t)}{1+a(t){\hat{X}}_{I}(x,t)+e(t)\tilde{Y}(x,t)}\right],\\ \frac{\partial \hat{Y}(x,t)}{\partial t}-D_{3}(t)\Delta \hat{Y}(x,t)\leq \hat{Y}(x,t)\left[-r_{2}(t)-d(t)\hat{Y}(x,t)+\frac{\gamma (t){\hat{X}}_{I}(x,t)}{1+a(t){\hat{X}}_{I}(x,t)+\hat{Y}(x,t)}\right], \end{array}$$

*and*

$$\begin{array}{l} \frac{\partial {\tilde{X}}_{S}(x,t)}{\partial n}\geq 0,\frac{\partial {\tilde{X}}_{I}(x,t)}{\partial n}\geq 0,\frac{\partial \tilde{Y}(x,t)}{\partial n}\geq 0,(x,t)\in \partial \Omega \times R^{+},\\ {\tilde{X}}_{S}(x,0)\geq \eta_{S0}(x),{\tilde{X}}_{I}(x,0)\geq \eta_{I0}(x),\tilde{Y}(x,0)\geq \eta_{Y0}(x),x\in \Omega ,\\ \frac{\partial {\hat{X}}_{S}(x,t)}{\partial n}\leq 0,\frac{\partial {\hat{X}}_{I}(x,t)}{\partial n}\leq 0,\frac{\partial \hat{Y}(x,t)}{\partial n}\leq 0,(x,t)\in \partial \Omega \times R^{+},\\ {\hat{X}}_{S}(x,0)\leq \eta_{S0}(x),{\hat{X}}_{I}(x,0)\leq \eta_{I0}(x),\hat{Y}(x,0)\leq \eta_{Y0}(x),x\in \Omega , \end{array}$$

*we called* $\tilde{U}(x,t),\hat{U}(x,t)$ *are a pair of ordered upper and lower solutions for model (2)–(3).*

**Definition 2.** 
*Suppose that there exist seven positive real numbers $Q_{i},q_{i},\;(i=1,2,3)$ and T, such that*

$$Q_{1}\geq X_{S}(x,t)\geq q_{1},\;Q_{2}\geq X_{I}(x,t)\geq q_{2},\;Q_{3}\geq Y(x,t)\geq q_{3},\;\mathrm{as}(x,t)\in \bar{\Omega }\times [T,+\infty )$$

*for each positive solution* $\left(X_{S}(x,t),X_{I}(x,t),Y(x,t)\right)$*of model (2)–(3) with any positive initials, then model (2)–(3) is called uniformity permanent. Similarly, we can define the persistence of the reduced model (ODE model without diffusion) corresponding to model (2)–(3).*

**Definition 3.** 
*If a smooth function *$V(t)=(X_{S}(t),X_{I}(t),Y(t))$*satisfies model (2)–(3) in $R^{+}$, and every component of V(t) is the ω-periodic function, we know that V(t) is a spatial homogeneity periodic solution for model (2)–(3), which is denoted as V(t,ω)*.

**Definition 4.** 
*For any non-negative smooth initial data $V(x,0)=(\eta_{S0}(x,0),\eta_{I0}(x,0),\eta_{Y0}(x,0))\geq 0$ and V(x,0)≡0, x∈Ω, if there exists a unique positive solution $V(x,t)=(X_{S}(x,t),X_{I}(x,t),Y(x,t))$ for model (2)–(3), and*

$$\lim_{t\to \infty }(V(x,t)-V(t,\omega ))=0,\;\mathrm{uniformly}\;\mathrm{for}\;x\in \bar{\Omega },$$

*we know that the spatial homogeneity periodic solution *V(t,ω) *is globally asymptotically stable.*

*Clearly, the reaction functions in model (3) satisfy mixed monotonicity from the non-negativity of ecosystem solutions. Moreover, the model presented in this paper is a special case of a model in the literature [40]. When the time delay in the model from [40] is set to 0 and the reaction function exhibits mixed monotonicity, the model in [40] reduces to the model in this paper. Therefore, based on [40], we obtain the following result.*

**Lemma 1.** 
*Suppose that $\tilde{U}(x,t),\hat{U}(x,t)$ are a pair of ordered upper and lower solutions for model (1)–(2), then there exists a global solution U(x,t) for model (2)–(3) such that*

$$\tilde{U}(x,t)\geq U(x,t)\geq \hat{U}(x,t),\;(x,t)\in \bar{\Omega }\times R^{+}.$$

**Lemma 2 ([41]).** 
*If the function $f(t):\;R^{+}\to R$ is uniformly continuous, and the limit $\underset{t\to \infty }{\lim}\int_{0}^{t}f(s)ds$ exists and is finite, then* $\underset{t\to +\infty }{\lim}f(t)=0.$

**Lemma 3 ([42]).** 
*Suppose that* $V\subset R_{n}$ *is compact and convex and the mapping* φ:V→V *is continuous, then there exists* $x^{*}\in V$ *such that *$\varphi (x^{*})=x^{*}$.

We now turn to the analysis of the reduced model of (2)–(3). When the population density in model (3)–(4) becomes independent of the spatial variable, model (2)–(3) will be reduced to the following model

(4) $$\left\{\begin{array}{l} \frac{dX_{S}(t)}{dt}=X_{S}(t)\left[r_{1}(t)-b(t)X_{S}(t)-(b(t)+\beta (t))X_{I}(t)\right],\;t\in R^{+},\\ \frac{dX_{I}(t)}{dt}=X_{I}(t)\left[-\delta (t)-c(t)X_{I}(t)+\psi (t)X_{S}(t)-\frac{\alpha (t)Y(t)}{1+a(t)X_{I}(t)+e(t)Y(t)}\right],\;t\in R^{+},\\ \frac{dY(t)}{dt}=Y(t)\left[-r_{2}(t)-d(t)Y(t)+\frac{\gamma (t)X_{I}(t)}{1+a(t)X_{I}(t)+e(t)Y(t)}\right],\;t\in R^{+}, \end{array}\right.$$

with the initial conditions

(5) $$X_{S}(0)=\eta_{X0}(0)>0,\;X_{I}(0)=\eta_{I0}(0)>0,Y(0)=\eta_{Y0}(0)>0.$$

Here, the parameters in model (4)–(5) are identical to those in model (2)–(3).

**Theorem 1.** 
*For any given initial values $(\eta_{S0}(0),\eta_{I0}(0),\eta_{Y0}(0))\in R_{+}^{3},$ the eco-epidemiological model (4)–(5) has a unique, positive, and global solution.*

**Proof.**  Since all the parameters in model (4) are continuous, positive, and bounded functions, it follows that the model satisfies the local Lipschitz condition in the space $R_{+}^{3}$. Thus, model (4)–(5) admits a unique local solution, denoted by $X_{S}(t),\;X_{I}(t)$ and Y(t), in some interval [0, *T*) according to the classical existence and uniqueness theorem for ordinary differential equations. Based on the practical significance of ecosystems, it is evident that the solutions to model (4) are non-negative. We now show that for any positive initial values, this solution remains positive and can be extended to all t≥0.

As indicated by the first equation in model (4), when $X_{S}(t)\geq 0,\;X_{I}(t)\geq 0$ and Y(t)≥0, the sign of its right-hand side is determined solely by

$$\Delta_{1}(t)≜r_{1}(t)-b(t)(X_{S}(t)+X_{I}(t))-\beta (t)X_{I}(t).$$

In the case where the initial values yield $\Delta_{1}(0)\geq 0$, the growth rate of $X_{S}(t)$ remains positive in some interval [0, *T*_1_) according to the local sign preservation theorem of continuous functions. Given the positivity of the initial value $\eta_{S0}(0)$, $X_{S}(t)$ is therefore guaranteed to stay positive in the interval [0, *T*_1_). Conversely, if the initial values lead to $\Delta_{1}(0)<0$, the growth rate of $X_{S}(t)$ becomes negative in some interval [0, *T*_1_*), and due to the positive initial value, the susceptible prey population $X_{S}(t)$ will continuously decrease. Based on the interaction mechanisms among populations within the ecosystem and the continuity of population dynamics, the infected prey population $X_{I}(t)$ will consequently decrease. Given that $r_{1}(t)>0$, as both $X_{S}(t)$ and $X_{I}(t)$ decline, the term $\Delta_{1}(t)$ will eventually become positive. This, in turn, causes the population of $X_{S}(t)$ to increase before it reaches zero. In summary, regardless of whether the initial values yield $\Delta_{1}(0)\geq 0$ or $\Delta_{1}(0)<0$, $X_{S}(t)$ maintains a positive value in some interval [0, *T*_1_**).

Similarly, as indicated by the second equation in model (3), when $X_{S}(t)\geq 0,\;X_{I}(t)\geq 0$ and Y(t)≥0, the sign of its right-hand side depends on

$$\Delta_{2}(t)≜-\delta (t)-c(t)X_{I}(t)+\psi (t)X_{S}(t)-\frac{\alpha (t)Y(t)}{1+a(t)X_{I}(t)+e(t)Y(t)}.$$

In the case where the initial values yield $\Delta_{2}(0)\geq 0$, the rate of change in $X_{I}(t)$ remains positive in some interval [0, *T*_2_) according to the local sign preservation theorem of continuous functions. Given the positivity of the initial value $\eta_{I0}(0)$, $X_{I}(t)$ will remain positive in the interval [0, *T*_2_). Conversely, if the initial values lead to $\Delta_{2}(0)<0$, then the rate of change in $X_{I}(t)$ is negative in some interval [0, *T*_2_*), and due to the positive initial value, the infected prey population $X_{I}(t)$ will continuously decrease. Based on the interaction mechanisms among populations within the ecosystem and the continuity of population dynamics, the susceptible prey population $X_{S}(t)$ will increase due to the reduction in infected prey population. At the same time, the predator population Y(t) will decrease due to the reduction in infected prey population. As $X_{I}(t)$ and Y(t) decrease and $X_{S}(t)$ increases, eventually $\Delta_{2}(t)$ will become positive (since ψ(t)>0). This, in turn, causes the population of $X_{I}(t)$ to increase before it reaches zero. In summary, regardless of whether the initial values make $\Delta_{2}(0)\geq 0$ or $\Delta_{2}(0)<0$, $X_{I}(t)$ maintains a positive value in some interval [0, *T*_2_**).

Furthermore, as indicated by the third equation in model (4), when $X_{S}(t)\geq 0,\;X_{I}(t)\geq 0$ and Y(t)≥0, the sign of its right-hand side depends on

$$\Delta_{3}(t)≜-r_{2}(t)-d(t)Y(t)+\frac{\gamma (t)X_{I}(t)}{1+a(t)X_{I}(t)+e(t)Y(t)}.$$

In the case where the initial values yield $\Delta_{3}(0)\geq 0$, the rate of change in Y(t) remains positive in some interval [0, *T*_3_) according to the local sign preservation theorem of continuous functions. Since the initial value $\mu_{Y0}(0)>0$, Y(t) will remain positive in the interval [0, *T*_3_). If the initial values result in $\Delta_{3}(0)<0$, then the rate of change in Y(t) becomes negative in some interval [0, *T*_3_*), and due to the positive initial value, the predator population Y(t) will continuously decrease. Based on the interaction mechanisms among populations within the ecosystem and the continuity of population dynamics, the infected prey population $X_{I}(t)$ will increase due to the reduction in the predator population. As Y(t) decreases and $X_{I}(t)$ increases, eventually $\Delta_{3}(t)$ will become positive (since γ(t)>0), causing the population of Y(t) to increase before decreasing to zero. In summary, regardless of whether the initial values make $\Delta_{3}(0)>0$ or $\Delta_{3}(0)<0$, Y(t) maintains a positive value in some interval [0, *T*_3_**).

Finally, since the unique local solution remains positive throughout its existence, and given that resource limitations prevent the ecosystem’s solution from reaching positive infinity, it follows from the continuation theorem for solutions of ordinary differential equations that this local solution can be extended to the entire time domain [0,+∞) while preserving positivity. Therefore, for any positive initial values, the solutions of the eco-epidemiological model (4)–(5) remain positive for all t≥0. ☐

**Remark 3.** 
*According to Theorem 1, for any positive initial values, model (3)–(4) has a unique positive global solution. Even if the solution decays exponentially to 0, it remains positive.*

**Theorem 2.** 
*For any given positive initial values $(\eta_{S0}(x,0),\eta_{I0}(x,0),\eta_{Y0}(x,0)),$ model (2)–(3) has a positive and global solution $(X_{S}(x,t),X_{I}(x,t),Y(x,t)),\;(x,t)\in \bar{\Omega }\times R_{+}$*.

**Proof.**  Set $l_{S}=\underset{x\in \bar{\Omega }}{\min}\eta_{S0}(x,0),\;l_{I}=\underset{x\in \bar{\Omega }}{\min}\eta_{I0}(x,0),\;l_{Y}=\underset{x\in \bar{\Omega }}{\min}\eta_{Y0}(x,0),\;$ $r_{S}=\underset{x\in \bar{\Omega }}{\max}\eta_{S0}(x,0),\;r_{I}=\underset{x\in \bar{\Omega }}{\max}\eta_{I0}(x,0)$, $r_{Y}=\underset{x\in \bar{\Omega }}{\max}\eta_{Y0}(x,0),$ then $0<l_{S}\leq \eta_{S0}(x,0)\leq r_{S},\;0<l_{I}\leq \eta_{I0}(x,0)\leq r_{I},\;0<l_{Y}\leq \eta_{Y0}(x,0)\leq r_{Y}.$ Let $\left({\tilde{X}}_{S}(t),{\tilde{X}}_{I}(t),{\tilde{X}}_{Y}(t)\right)$ and $\left({\hat{X}}_{S}(t),{\hat{X}}_{I}(t),{\hat{X}}_{Y}(t)\right)$ are the solutions for ordinary differential Equation (4) subject to initial values $\left(r_{S},r_{I},r_{Y}\right)$ and $\left(l_{S},l_{I},l_{Y}\right)$, respectively. Because the solution of model (4)–(5) is also a solution of model (2)–(3), from Definition 1 there exist a pair of ordered upper and lower solutions $\left({\tilde{X}}_{S}(t),{\tilde{X}}_{I}(t),{\tilde{X}}_{Y}(t)\right)$ and $\left({\hat{X}}_{S}(t),{\hat{X}}_{I}(t),{\hat{X}}_{Y}(t)\right)$ for model (2)–(3). Therefore, from Lemma 1 and Theorem 1, model (2)–(3) has a global solution $(X_{S}(x,t),X_{I}(x,t),X_{Y}(x,t)),\;(x,t)\in \bar{\Omega }\times R^{+},$ which satisfies

$$(0,0,0)<({\hat{X}}_{S}(t),{\hat{X}}_{I}(t),{\hat{X}}_{Y}(t))\leq (X_{S}(x,t),X_{I}(x,t),X_{Y}(x,t))\leq ({\tilde{X}}_{S}(t),{\tilde{X}}_{I}(t),{\tilde{X}}_{Y}(t)),\;(x,t)\in \bar{\Omega }\times R^{+}.$$

This ends the proof of Theorem 2. ☐

Suppose that φ(x) is a ω-periodic function in $R^{+}$, we denote


$$\varphi^{m}=\sup\{\varphi (x),x\in R^{+}\},\;\varphi^{l}=\inf\left\{\varphi (x),x\in R^{+}.\right\}$$

For the ordinary differential Equation (4), we let

$$\begin{array}{l} \;M_{1}^{*}=\frac{r_{1}^{m}}{b^{l}},M_{2}^{*}=\frac{\psi^{m}M_{1}-\delta^{l}}{c^{l}},M_{3}^{*}=\frac{\gamma_{1}^{m}M_{2}-r_{2}^{l}}{d^{l}},\\ m_{1}^{*}=\frac{r_{1}^{l}-(b^{m}+\beta^{m})M_{2}}{b^{m}},\;m_{2}^{*}=\frac{\psi^{l}m_{1}-\alpha^{m}M_{3}-\delta^{m}}{c^{m}},\\ \;\;\;\;m_{3}^{*}=\frac{\gamma^{l}m_{2}}{d^{m}(1+a^{m}M_{2}+e^{m}M_{3})}-\frac{r_{2}^{m}}{d^{m}}, \end{array}$$

where $M_{i},\;m_{i},\;(i=1,2,3)$ are appropriate positive real numbers such that


(6) $$0<m_{i}<m_{i}^{*}<M_{i}^{*}<M_{i},\;i=1,\;2,3\;.$$

**Theorem 3.** 
*If it holds that*

$$\begin{array}{l} (H_{1})\;\psi^{m}M_{1}>\delta^{l},\;(H_{2})\;\gamma_{1}^{m}M_{2}>r_{2}^{l},\\ \;(H_{3})\;r_{1}^{l}>(b^{m}+\beta^{m})M_{2},\;\;(H_{4})\;\psi^{l}m_{1}>\alpha^{m}M_{3}+\delta^{m},\\ \;(H_{5})\;\frac{\gamma^{l}m_{2}}{d^{m}(1+a^{m}M_{2}+e^{m}M_{3})}>\frac{r_{2}^{m}}{d^{m}}, \end{array}$$

*then the ordinary differential Equation (4) is permanent.*

**Proof.**  From the first equation of model (4), we have

$$\begin{array}{ccc} \frac{dX_{S}(t)}{dt} & = & X_{S}(t)[r_{1}(t)-b(t)X_{S}(t)-(b(t)+\beta (t))X_{I}(t)]\\ & \leq & X_{S}(t)[r_{1}(t)-b(t)X_{S}(t)]\leq X_{S}(t)[r_{1}^{m}-b^{l}X_{S}(t)]=b^{l}X_{S}(t)[-X_{S}(t)+\frac{r_{1}^{m}}{b^{l}}]. \end{array}$$

Using the comparison theorem of ordinary differential equations, the following can be established:

(1) If $0<X_{S}(0)<M_{1}$, then $X_{S}(t)\leq M_{1}$ for any t≥0.
(2) If $X_{S}(0)\geq M_{1}$, then $X_{S}(t)\leq M_{1}$ for a sufficiently large t. Otherwise, if $X_{S}(t)>M_{1}$, then there exists some α>0 such that $X_{S}(t)\geq M_{1}^{*}+\alpha$. Furthermore, we have

$${\left.\frac{dX_{S}(t)}{dt}\right|}_{x_{1}(t)>M_{1}}\leq b^{l}X_{S}(t)[\frac{r_{1}^{m}}{b^{l}}-X_{S}(t)]=b^{l}X_{S}(t)[M_{1}^{*}-X_{S}(t)]<-\alpha b^{l}X_{S}(t),$$

and, thus, it follows that

$$X_{S}(t)<X_{S}(t_{0})\exp(-\alpha b^{l}(t-t_{0}))\to 0.\;\mathrm{for}\;\mathrm{any}\;t\to +\infty .$$

The above inequality contradicts $X_{S}(t)>M_{1}$. Hence, we may choose a sufficiently large $T_{1}>0$ such that

(7) $$X_{S}(t)\leq M_{1}\;\mathrm{for}\;\mathrm{any}\;t>T_{1}.$$

Similarly, considering the second equation in (4), and using (7), it follows that

$$\begin{array}{cc} \frac{dX_{I}(t)}{dt} & =X_{I}(t)[-\delta (t)-c(t)X_{I}(t)+\psi (t)X_{S}(t)-\frac{\alpha (t)Y(t)}{1+a(t)X_{I}(t)+e(t)Y(t)}]\\ & \leq X_{I}(t)[-\delta (t)-c(t)X_{I}(t)+\psi (t)X_{S}(t)]\\ & \leq X_{I}(t)[-\delta^{l}-c^{l}X_{I}(t)+\psi^{m}M_{1}]\\ & =c^{l}X_{I}(t)[-X_{I}(t)+\frac{\psi^{m}M_{1}-\delta^{l}}{c^{l}}]. \end{array}$$

Using the same analysis as in the first equation, there will be a large enough $T_{2}>0$ so that

(8) $$X_{I}(t)<M_{2}\;\mathrm{for}\;\mathrm{any}\;t>T_{2}.$$

Based on the third equation of model (4), and using (8), it follows that

$$\begin{array}{cc} \frac{dY(t)}{dt} & =Y(t)[-r_{2}(t)-d(t)Y(t)+\frac{\gamma (t)X_{I}(t)}{1+a(t)X_{I}(t)+e(t)Y(t)}]\\ & \leq Y(t)[-r_{2}(t)-d(t)Y(t)+\gamma (t)X_{I}(t)]\\ & \leq Y(t)[-r_{2}^{l}-d^{l}Y(t)+\gamma_{1}^{m}M_{2}]\\ & =d^{l}Y(t)[-Y(t)+\frac{\gamma_{1}^{m}M_{2}-r_{2}^{l}}{d^{l}}]. \end{array}$$

Consistent with the method outlined above, we find the following:

(3) If $0<Y(0)<M_{3}$, then $Y(t)\leq M_{3}$ for any t≥0.
(4) If $Y(0)\geq M_{3}$, then $Y(t)\leq M_{3}$ for a sufficiently large t.

Therefore, it holds that there is a sufficiently large $T_{3}>0$ such that

(9) $$Y(t)\leq M_{3}\;\mathrm{for}\;\mathrm{any}\;t>T_{3}.$$

We now establish that $\left(X_{S}(t),X_{I}(t),X_{Y}(t)\right)$ is bounded below by a positive constant. From the first equation in (3), and using (7), it follows that

$$\begin{array}{cc} \frac{dX_{S}(t)}{dt} & =X_{S}(t)[r_{1}(t)-b(t)X_{S}(t)-(b(t)+\beta (t))X_{I}(t)]\\ & \geq X_{S}(t)[r_{1}^{l}-b^{m}X_{S}(t)-(b^{m}+\beta^{m})M_{2}]\\ & =b^{m}X_{S}(t)[-X_{S}(t)+\frac{r_{1}^{l}-(b^{m}+\beta^{m})M_{2}}{b^{m}}]. \end{array}$$

By employing the comparison theorem for ordinary differential equations, we derive the following:

(5) If $m_{1}<X_{S}(0)$, then $m_{1}<X_{S}(t)$ for any t≥0.
(6) If $0<X_{S}(0)\leq m_{1}$, then $m_{1}<X_{S}(t)$ for a sufficiently large t. Otherwise, if $X_{S}(t)<m_{1},$ then there is β>0 such that $X_{S}(t)\leq m_{1}^{*}-\beta .$ Hence, it follows that

$$\begin{array}{l} {\left.\frac{dX_{S}(t)}{dt}\right|}_{X_{S}(t)<m_{1}}\geq b^{m}X_{S}(t)[\frac{r_{1}^{l}-(b^{m}+\beta^{m})M_{2}}{b^{m}}-X_{S}(t)]\\ \;\;\;\;\;\;\;\;\geq b^{m}X_{S}(t)[-X_{S}(t)+m_{1}^{*}]\geq b^{m}\beta X_{S}(t). \end{array}$$

Thus, we obtain

$$X_{S}(t)\geq X_{S}(t_{0})\exp(b^{m}\beta (t-t_{0}))\to +\infty \mathrm{for}\;\mathrm{any}\;t\to +\infty .$$

The above inequality contradicts $X_{S}(t)<m_{1}.$ Hence, we may choose a sufficiently large ${T^{\prime }}_{1}>0$ such that

(10) $$X_{S}(t)\geq m_{1}\;\mathrm{as}\;t>T_{1}^{\prime }.$$

Analogously, considering the second equation in (4), and using (9) and (10), it follows that

$$\begin{array}{cc} \frac{dX_{I}(t)}{dt} & =X_{I}(t)[-\delta (t)-c(t)X_{I}(t)+\psi (t)X_{S}(t)-\frac{\alpha (t)Y(t)}{1+a(t)X_{I}(t)+e(t)Y(t)}]\\ & \geq X_{I}(t)[-\delta (t)-c(t)X_{I}(t)+\psi (t)X_{S}(t)-\alpha (t)Y(t)]\\ & \geq X_{I}(t)[-\delta^{m}-c^{m}X_{I}(t)+\psi^{l}m_{1}-\alpha^{m}M_{3}]\\ & \geq c^{m}X_{I}(t)[-X_{I}(t)+\frac{\psi^{l}m_{1}-\alpha^{m}M_{3}-\delta^{m}}{c^{m}}]. \end{array}$$

Using the comparison theorem and the same analysis method above, we know that there exists a large enough $T_{2}^{\prime }>0$ so that

(11) $$X_{I}(t)>m_{2}\;\mathrm{for}\;\mathrm{any}\;t>T_{2}^{\prime }\;.$$

Based on the third equation in model (4), and from (8), (9), and (11), we achieve

$$\begin{array}{cc} \frac{dY(t)}{dt} & =Y(t)[-r_{2}(t)-d(t)Y(t)+\frac{\gamma (t)X_{I}(t)}{1+a(t)X_{I}(t)+e(t)Y(t)}]\\ & \geq Y(t)[-r_{2}^{m}-d^{m}Y(t)+\frac{\gamma^{l}m_{2}}{1+a^{m}M_{2}+e^{m}M_{3}}]\\ & \geq d^{m}Y(t)[-Y(t)+\frac{\gamma^{l}m_{2}}{d^{m}(1+a^{m}M_{2}+e^{m}M_{3})}-\frac{r_{2}^{m}}{d^{m}}]. \end{array}$$

Using the comparison theorem and the same analysis method above, it follows that

(7) If $m_{3}<Y(0)$, then $m_{3}\leq Y(t)$ for any t≥0.
(8) If $Y(0)\geq m_{3}$, then $m_{3}\leq Y(t)$ for a sufficiently large t.

Thus, we may choose a enough large $T_{3}^{\prime }>0$ such that

(12) $$Y(t)>m_{3}\;\mathrm{for}\;\mathrm{any}\;t>T_{3}^{\prime }.$$

From inequalities (7)–(12), and by setting $T=\underset{1\leq i\leq 3}{\max}\left\{T_{i}^{,}{T^{\prime }}_{i}\right\}$, it follows that $m_{1}\leq X_{S}(t)\leq M_{1},\;$ $m_{2}\leq X_{I}(t)\leq M_{2},\;$ $m_{3}\leq Y(t)\leq M_{3},$ as t>T for any solution $\left(X_{S}(t),X_{I}(t),Y(t)\right)$ of model (4) with any positive initial values. This completes the proof of Theorem 3. ☐

**Theorem 4.** 
*If it follows that*

$$\begin{array}{l} (H_{1})\;\psi^{m}M_{1}>\delta^{l},\;(H_{2})\;\gamma_{1}^{m}M_{2}>r_{2}^{l},\\ \;(H_{3})\;r_{1}^{l}>(b^{m}+\beta^{m})M_{2},\;\;(H_{4})\;\psi^{l}m_{1}>\alpha^{m}M_{3}+\delta^{m},\\ \;(H_{5})\;\frac{\gamma^{l}m_{2}}{d^{m}(1+a^{m}M_{2}+e^{m}M_{3})}>\frac{r_{2}^{m}}{d^{m}}. \end{array}$$

*then the partial differential Equation (3) is permanent.*

**Proof.**  This can be proved by Theorem 3 using a proof method similar to that of Theorem 2. This is omitted here. ☐

## 4. Existence and Stability of the Spatially Homogeneous Periodic Solution

In this section, the existence and global asymptotic stability of a spatially homogeneous periodic solution are established for the nonautonomous *ω*-periodic reaction–diffusion predator–prey model with disease in the prey and Beddington–DeAngelis functional response, as described by model (2)–(3). By applying the method of upper and lower solutions for parabolic partial differential equations and Lyapunov stability theory, we derive a set of easily verifiable sufficient conditions that ensure these dynamical properties.

**Theorem 5.** 
Provided that all assumptions $\left(H_{1})-(H_{5}\right)$ hold for the parameters in model (2)–(3), we obtain that the model possesses a strictly positive, spatially homogeneous ω-periodic solution $U(t)=\left(X_{S}^{*}(t),X_{I}^{*}(t),Y^{*}(t)\right)$.

**Proof.**  By virtue of the existence and uniqueness theorem for solutions of ordinary differential equations, which ensures a well-defined flow map, one may define the Poincaré operator $\varphi :\;R_{+}^{3}\to R_{+}^{3}$ as follows:

$$\varphi (U_{0})=U(t,\;\omega ,\;t_{0},\;U_{0}),$$

where $U(t,\omega ,t_{0},U_{0})=(X_{S}(t),X_{I}(t),Y(t))$ is a positive solution of model (4) subject to the initial conditions $U_{0}=(X_{S}(t_{0}),X_{I}(t_{0}),Y(t_{0}))$. Moreover, if we define

$$S=\left\{(X_{S},X_{I},Y)\in R_{+}^{3}\left|m_{1}\leq X_{S}\leq M_{1}\right.,\;m_{2}\leq X_{I}\leq M_{2},\;m_{3}\leq Y\leq M_{3}\right\},$$

then it follows that $S\subset R_{+}^{3}$ is a compact and convex set. By Theorem 3 and the continuity of the solution to model (3) under the given initial conditions, the mapping φ is continuous from S into itself. Furthermore, it follows from Lemma 3 that model (4) admits a positive ω-periodic solution $U(t)=(X_{S}^{*}(t),X_{I}^{*}(t),$ $Y^{*}(t)),\;t\in R^{+},$ which corresponds to a strictly positive spatially homogeneous ω-periodic solution for the original model (2)–(3). This completes the proof of Theorem 5. ☐

**Theorem 6.** 
*Suppose that the nonautonomous reaction-diffusive ω-periodic model (2)–(3) satisfies the assumptions $\left(H_{1})-(H_{5}\right)$ and the following additional conditions*

$$\begin{array}{l} (H_{6})\;b^{l}-\psi^{m}>0,\;\\ (H_{7})\;c^{l}-b^{m}-\beta^{m}-\frac{a^{m}\alpha^{m}M_{3}+\gamma^{m}(1+e^{m}M_{3})}{{\left(1+a^{l}m_{2}+e^{l}m_{3}\right)}^{2}}>0,\;\\ (H_{8})\;d^{l}-\frac{\alpha^{m}+a^{m}\alpha^{m}M_{2}-\gamma^{l}e^{l}m_{2}}{{\left(1+a^{l}m_{2}+e^{l}m_{3}\right)}^{2}}>0, \end{array}$$

*then the model admits a strictly positive spatially homogeneous periodic solution* $(X_{S}^{*}(t),X_{I}^{*}(t),Y^{*}(t)).$ *Moreover, this solution is globally asymptotically stable. That is, for any positive initial values, the solution * $\left(X_{S}(x,t),X_{I}(x,t),Y(x,t)\right)$ *of the nonautonomous ω-periodic model (2)–(3) satisfies*

(13) $$\underset{t\to \infty }{\lim}(X_{S}(x,t)-X_{S}^{*}(t))=\underset{t\to \infty }{\lim}(X_{I}(x,t)-X_{I}^{*}(t))=\underset{t\to \infty }{\lim}(Y(x,t)-Y^{*}(t))=0,\;\mathrm{uniformly}\;\mathrm{for}\;x\in \bar{\Omega }.$$

**Proof.**  Following the existence results of Theorem 5, we focus on proving stability. According to Theorem 2, for any positive initial value $(\eta_{S0}(x,0),\eta_{I0}(x,0),\eta_{Y0}(x,0)),$ model (2)–(3) has a positive and globally solution $(X_{S}(x,t),X_{I}(x,t),X_{Y}(x,t)),\;(x,t)\in \bar{\Omega }\times R^{+},$ which satisfies

$$({\hat{X}}_{S}(t),{\hat{X}}_{I}(t),{\hat{X}}_{Y}(t))\leq (X_{S}(x,t),X_{I}(x,t),X_{Y}(x,t))\leq ({\tilde{X}}_{S}(t),{\tilde{X}}_{I}(t),{\tilde{X}}_{Y}(t)),$$

where $\left({\tilde{X}}_{S}(t),{\tilde{X}}_{I}(t),{\tilde{X}}_{Y}(t)\right)$ and $\left({\hat{X}}_{S}(t),{\hat{X}}_{I}(t),{\hat{X}}_{Y}(t)\right)$ are the solutions for model (4)–(5).

To establish (13), according to the squeeze theorem it suffices to prove

(14) $$\begin{array}{l} \underset{t\to \infty }{\lim}({\tilde{X}}_{S}(t)-X_{S}^{*}(t))=\underset{t\to \infty }{\lim}({\tilde{X}}_{I}(t)-X_{I}^{*}(t))=\underset{t\to \infty }{\lim}(\tilde{Y}(t)-Y^{*}(t))=0,\\ \underset{t\to \infty }{\lim}({\hat{X}}_{S}(t)-X_{S}^{*}(t))=\underset{t\to \infty }{\lim}({\hat{X}}_{I}(t)-X_{I}^{*}(t))=\underset{t\to \infty }{\lim}(\hat{Y}(t)-Y^{*}(t))=0. \end{array}$$

This is achieved by demonstrating that any solution $\left(X_{S}(t),X_{I}(t),Y(t)\right)$ for model (4) with an arbitrary positive initial value satisfies

(15) $$\underset{t\to \infty }{\lim}(X_{S}(t)-X_{S}^{*}(t))=\underset{t\to \infty }{\lim}(X_{I}(t)-X_{I}^{*}(t))=\underset{t\to \infty }{\lim}(Y(t)-Y^{*}(t))=0.$$

Applying Theorem 3 yields the existence of positive constants $M_{i},m_{i},(i=1,2,3)$ and T such that

$$m_{1}\leq X_{S}(t)\leq M_{1},\;m_{2}\leq X_{I}(t)\leq M_{2},m_{3}\leq Y(t)\leq M_{3}\mathrm{for}\;\mathrm{all}\;t>T.$$

Consider the Lyapunov function

$$V(t)=\left|\ln X_{S}(t)-\ln X_{S}^{*}(t)\right|+\left|\ln X_{I}(t)-\ln X_{I}^{*}(t)\right|+\left|\ln Y(t)-\ln Y^{*}(t)\right|.$$

Let $D^{+}V(t)$ represent its right-hand derivative along solutions of model (4). Then, we obtain

$$\begin{array}{ll} D^{+}V(t) & =D^{+}[\left|\ln X_{S}(t)-\ln X_{S}^{*}(t)\right|]+D^{+}[\left|\ln X_{I}(t)-\ln X_{I}^{*}(t)\right|]+D^{+}[\left|\ln Y(t)-\ln Y^{*}(t)\right|]\\ & \leq \mathrm{sgn}\left\{X_{S}(t)-X_{S}^{*}(t)\right\}[-b(t)(X_{S}(t)-X_{S}^{*}(t))-(b(t)+\beta (t))(X_{I}(t)-X_{I}^{*}(t))]\\ & \;\;+\mathrm{sgn}\{X_{I}(t)-X_{I}^{*}(t)\}[-c(t)(X_{I}(t)-X_{I}^{*}(t))+\psi (t)(X_{S}(t)-X_{S}^{*}(t))\;\\ & \;\;-(\frac{\alpha (t)Y(t)}{1+a(t)X_{I}(t)+e(t)Y(t)}-\frac{\alpha (t)Y^{*}(t)}{1+a(t)X_{1}^{*}(t)+e(t)Y^{*}(t)})]\\ & \;\;+\mathrm{sgn}\{Y(t)-Y^{*}(t)\}[-d(t)(Y(t)-Y^{*}(t))+(\frac{\gamma (t)X_{I}(t)}{1+a(t)X_{I}(t)+e(t)Y(t)}\\ & \;\;-\frac{\gamma (t)X_{I}^{*}(t)}{1+a(t)X_{I}^{*}(t)+e(t)Y^{*}(t)})]\\ & =\mathrm{sgn}\left\{X_{S}(t)-X_{S}^{*}(t)\right\}[-b(t)(X_{S}(t)-X_{S}^{*}(t))-(b(t)+\beta (t))(X_{I}(t)-X_{I}^{*}(t))]\\ & \;\;+\mathrm{sgn}\{X_{I}(t)-X_{I}^{*}(t)\}[-c(t)(X_{I}(t)-X_{I}^{*}(t))+\psi (t)(X_{S}(t)-X_{S}^{*}(t))\;\\ & \;\;-\frac{\alpha (t)Y(t)(1+a(t)X_{1}^{*}(t)+e(t)Y^{*}(t))-\alpha (t)Y^{*}(t)(1+a(t)X_{I}(t)+e(t)Y(t))}{\left(1+a(t)X_{I}(t)+e(t)Y(t))(1+a(t)X_{1}^{*}(t)+e(t)Y^{*}(t)\right)}\\ & \;\;+\mathrm{sgn}\{Y(t)-Y^{*}(t)\}[-d(t)(Y(t)-Y^{*}(t))\\ & \;\;+\frac{\gamma (t)X_{I}(t)(1+a(t)X_{I}^{*}(t)+e(t)Y^{*}(t))-\gamma (t)X_{I}^{*}(t)(1+a(t)X_{I}(t)+e(t)Y(t))}{\left(1+a(t)X_{I}(t)+e(t)Y(t))(1+a(t)X_{I}^{*}(t)+e(t)Y^{*}(t)\right)}] \end{array}$$

$$\begin{array}{ll} & =\mathrm{sgn}\left\{X_{S}(t)-X_{S}^{*}(t)\right\}[-b(t)(X_{S}(t)-X_{S}^{*}(t))-(b(t)+\beta (t))(X_{I}(t)-X_{I}^{*}(t))]\\ & \;\;+\mathrm{sgn}\{X_{I}(t)-X_{I}^{*}(t)\}[-c(t)(X_{I}(t)-X_{I}^{*}(t))+\psi (t)(X_{S}(t)-X_{S}^{*}(t))\;\\ & \;\;-\frac{\alpha (t)Y(t)+a(t)\alpha (t)X_{1}^{*}(t)Y(t)-\alpha (t)Y^{*}(t)-a(t)\alpha (t)Y^{*}(t)X_{I}(t)}{\left(1+a(t)X_{I}(t)+e(t)Y(t))(1+a(t)X_{1}^{*}(t)+e(t)Y^{*}(t)\right)}]\\ & \;\;+\mathrm{sgn}\{Y(t)-Y^{*}(t)\}[-d(t)(Y(t)-Y^{*}(t))\\ & \;\;+\frac{\gamma (t)X_{I}(t)+\gamma (t)e(t)X_{I}(t)Y^{*}(t)-\gamma (t)X_{I}^{*}(t)-\gamma (t)e(t)X_{I}^{*}(t)Y(t)}{\left(1+a(t)X_{I}(t)+e(t)Y(t))(1+a(t)X_{I}^{*}(t)+e(t)Y^{*}(t)\right)}]\\ & =\mathrm{sgn}\left\{X_{S}(t)-X_{S}^{*}(t)\right\}[-b(t)(X_{S}(t)-X_{S}^{*}(t))-(b(t)+\beta (t))(X_{I}(t)-X_{I}^{*}(t))]\\ & \;\;+\mathrm{sgn}\{X_{I}(t)-X_{I}^{*}(t)\}[-c(t)(X_{I}(t)-X_{I}^{*}(t))+\psi (t)(X_{S}(t)-X_{S}^{*}(t))\;\\ & \;\;-\frac{\alpha (t)(Y(t)-Y^{*}(t))+a(t)\alpha (t)X_{I}^{*}(t)(Y(t)-Y^{*}(t))-a(t)\alpha (t)Y^{*}(t)(X_{I}(t)-X_{1}^{*}(t))}{\left(1+a(t)X_{I}(t)+e(t)Y(t))(1+a(t)X_{1}^{*}(t)+e(t)Y^{*}(t)\right)}]\\ & \;\;+\mathrm{sgn}\{Y(t)-Y^{*}(t)\}[-d(t)(Y(t)-Y^{*}(t))\\ & \;\;+\frac{\gamma (t)(X_{I}(t)-X_{I}^{*}(t))+\gamma (t)e(t)Y^{*}(t)(X_{I}(t)-X_{I}^{*}(t))-\gamma (t)e(t)X_{I}^{*}(t)(Y(t)-Y^{*}(t))}{\left(1+a(t)X_{I}(t)+e(t)Y(t))(1+a(t)X_{I}^{*}(t)+e(t)Y^{*}(t)\right)}]\\ & \leq \left|X_{S}(t)-X_{S}^{*}(t)\right|[-b(t)+\psi (t)]\\ & \;\;+\left|X_{I}(t)-X_{I}^{*}(t)\right|[b(t)+\beta (t)-c(t)+\frac{a(t)\alpha (t)Y^{*}(t)+\gamma (t)(1+e(t)Y^{*}(t))}{\left(1+a(t)X_{I}(t)+e(t)Y(t))(1+a(t)X_{1}^{*}(t)+e(t)Y^{*}(t)\right)}]\\ & \;\;+\left|Y(t)-Y^{*}(t)\right|[-d(t)+\frac{\alpha (t)+a(t)\alpha (t)X_{1}^{*}(t)-\gamma (t)e(t)X_{I}^{*}(t)}{\left(1+a(t)X_{I}(t)+e(t)Y(t))(1+a(t)X_{1}^{*}(t)+e(t)Y^{*}(t)\right)}]\\ & \leq -\left|X_{S}(t)-X_{S}^{*}(t)\right|[b(t)-\psi (t)]\\ & \;\;-\left|X_{I}(t)-X_{I}^{*}(t)\right|[c(t)-b(t)-\beta (t)-\frac{a(t)\alpha (t)Y^{*}(t)+\gamma (t)(1+e(t)Y^{*}(t))}{\left(1+a(t)X_{I}(t)+e(t)Y(t))(1+a(t)X_{1}^{*}(t)+e(t)Y^{*}(t)\right)}]\\ & \;\;-\left|Y(t)-Y^{*}(t)\right|[d(t)-\frac{\alpha (t)+a(t)\alpha (t)X_{I}^{*}(t)-\gamma (t)e(t)X_{I}^{*}(t)}{\left(1+a(t)X_{I}(t)+e(t)Y(t))(1+a(t)X_{1}^{*}(t)+e(t)Y^{*}(t)\right)}]\\ & \leq -\left|X_{S}(t)-X_{S}^{*}(t)\right|[b^{l}-\psi^{m}]\\ & \;\;-\left|X_{I}(t)-X_{I}^{*}(t)\right|[c^{l}-b^{m}-\beta^{m}-\frac{a^{m}\alpha^{m}M_{3}+\gamma^{m}(1+e^{m}M_{3})}{{\left(1+a^{l}m_{2}+e^{l}m_{3}\right)}^{2}}]\\ & \;\;-\left|Y(t)-Y^{*}(t)\right|[d^{l}-\frac{\alpha^{m}+a^{m}\alpha^{m}M_{2}-\gamma^{l}e^{l}m_{2}}{{\left(1+a^{l}m_{2}+e^{l}m_{3}\right)}^{2}}]. \end{array}$$

Based on assumptions $\left(H_{6})-(H_{8}\right)$, we obtain that

$$\alpha =\min\{b^{l}-\psi^{m},c^{l}-b^{m}-\beta^{m}-\frac{a^{m}\alpha^{m}M_{3}+\gamma^{m}(1+e^{m}M_{3})}{{\left(1+a^{l}m_{2}+e^{l}m_{3}\right)}^{2}},\;d^{l}-\frac{\alpha^{m}+a^{m}\alpha^{m}M_{2}-\gamma^{l}e^{l}m_{2}}{{\left(1+a^{l}m_{2}+e^{l}m_{3}\right)}^{2}}\}>0.$$

Thus,

(16) $$D^{+}V(t)\leq -\alpha [\left|X_{S}(t)-X_{S}^{*}(t)\right|+\left|X_{I}(t)-X_{I}^{*}(t)\right|+\left|Y(t)-Y^{*}(t)\right|]$$

Integrating (16) from T to t ($T\geq t_{0}$), we can achieve that

$$V(t)+\alpha \int_{T}^{t}(\left|X_{S}(t)-X_{S}^{*}(t)\right|+\left|X_{I}(t)-X_{I}^{*}(t)\right|+\left|Y(t)-Y^{*}(t)\right|)ds\leq V(T)<+\infty .$$

Therefore,

(17) $$\int_{T}^{t}\left(\left|X_{S}(t)-X_{S}^{*}(t)\right|+\left|X_{I}(t)-X_{I}^{*}(t)\right|+\left|Y(t)-Y^{*}(t)\right|\right)ds\leq \frac{V(T)}{\alpha }<+\infty .$$

By means of (17), we have

$$(\left|X_{S}(t)-X_{S}^{*}(t)\right|+\left|X_{I}(t)-X_{I}^{*}(t)\right|+\left|Y(t)-Y^{*}(t)\right|)\in L^{1}(T,+\infty ).$$

Moreover, it follows that

$$\begin{array}{l} \left|X_{S}(t)-X_{S}^{*}(t)\right|\leq \left|X_{S}(t)\right|+\left|X_{S}^{*}(t)\right|,\\ \left|X_{I}(t)-X_{I}^{*}(t)\right|\leq \left|X_{I}(t)\right|+\left|X_{I}^{*}(t)\right|,\\ \;\left|Y(t)-Y^{*}(t)\right|\leq \left|Y(t)\right|+\left|Y^{*}(t)\right|, \end{array}$$

and

$$\begin{array}{l} \left|\frac{d(X_{S}(t)-X_{S}^{*}(t))}{dt}\right|\leq \left|\frac{dX_{S}(t)}{dt}\right|+\left|\frac{dX_{S}^{*}(t)}{dt}\right|,\\ \left|\frac{d(X_{I}(t)-X_{I}^{*}(t))}{dt}\right|\leq \left|\frac{dX_{I}(t)}{dt}\right|+\left|\frac{dX_{I}^{*}(t)}{dt}\right|,\\ \;\left|\frac{d(Y(t)-Y^{*}(t))}{dt}\right|\leq \left|\frac{dY(t)}{dt}\right|+\left|\frac{dY^{*}(t)}{dt}\right|. \end{array}$$

Thus, from the uniformity permanence of model (4), $\left(X_{S}(t)-X_{S}^{*}(t)),\;(X_{I}(t)-X_{I}^{*}(t)\right)$ $,\;(Y(t)-Y^{*}(t))$ and their derivatives are bounded on the interval [T,+∞). Hence, $\left(X_{S}(t)-X_{S}^{*}(t))+(X_{I}(t)-X_{I}^{*}(t)\right)$ $+(Y(t)-Y^{*}(t))$ is uniformly continuous. Since the absolute value of a uniformly continuous function must be continuous, $\left|X_{S}(t)-X_{S}^{*}(t)\right|+\left|X_{I}(t)-X_{I}^{*}(t)\right|+\left|Y(t)-Y^{*}(t)\right|$ is uniformly continuous. Based on Lemma 2, we obtain that

$$\underset{t\to \infty }{\lim}\left|X_{S}(t)-X_{S}^{*}(t)\right|=0,\underset{t\to \infty }{\lim}\left|X_{I}(t)-X_{I}^{*}(t)\right|=0,\underset{t\to \infty }{\lim}\left|Y(t)-Y^{*}(t)\right|=0.$$

Thus, the proof of Theorem 6 is completed. ☐

## 5. Numerical Example

To numerically validate the theoretical achievements of this paper, especially the global stability conclusion established in Theorem 6, we have meticulously planned and carried out a simulation study with specifically chosen parameters for in-depth analysis. In the nonautonomous predator–prey model that features prey infection and incorporates a Beddington–DeAngelis functional response (1)–(2), we set all coefficients as two-periodic functions. This arrangement is aimed at strictly satisfying the various hypotheses proposed in Theorem 6. This highly representative example not only clearly confirms the asymptotic behavior characteristics of the solution trajectories but also fully demonstrates the high degree of consistency between our rigorously derived analytical results and actual numerical observations.

**Example 1.**  *Consider the following reaction–diffusion* 2-*periodic predator–prey model with prey infection. In accordance with the hypotheses* $\left(H_{1})-(H_{8}\right)$ *of Theorem 6, and through analytical and numerical computations, we select a specific set of parameter values (as detailed in model (17)–(18)) under which the model exhibits the desired dynamical behavior. It is important to emphasize that this particular parameter selection is not unique; rather, it serves as one representative configuration that satisfies the required assumptions and facilitates numerical verification of the theoretical results.*

(18) $$\begin{array}{cc} \frac{\partial X_{S}(x,t)}{\partial t}-\Delta X_{S}(x,t) & =X_{S}(x,t)[(10+\sin\pi t)-(4.4+0.1\sin\pi t)X_{S}(x,t)\\ & \;\;-((4.4+0.1\sin\pi t)+(1.5+0.1\sin\pi t))X_{I}(x,t)],\\ \frac{\partial X_{I}(x,t)}{\partial t}-\Delta X_{I}(x,t) & =X_{I}(x,t)[-(2+0.01\sin\pi t)-(10.46+0.11\sin\pi t)X_{I}(x,t)\\ & \;\;+(1.49+0.99\sin\pi t)X_{S}(x,t)\\ & \;\;-\frac{\left(0.5+0.01\sin\pi t)Y(x,t\right)}{1+(0.007+0.01\sin\pi t)X_{I}(x,t)+(0.04+0.01\sin\pi t)Y(x,t)}],\\ \frac{\partial Y(x,t)}{\partial t}-\Delta Y(x,t) & =Y(x,t)[-(0.08+0.01\sin\pi t)-(3.85+0.01\sin\pi t)Y(x,t)\\ & \;\;+\frac{\left(3.65+0.02\sin\pi t)X_{I}(x,t\right)}{1+(0.007+0.01\sin\pi t)X_{I}(x,t)+(0.04+0.01\sin\pi t)Y(x,t)}], \end{array}$$

subject to the following Neumann boundary conditions and initial values:

(19) $$\frac{\partial X_{S}}{\partial n}=\frac{\partial X_{I}}{\partial n}=\frac{\partial Y}{\partial n}=0,\;t>0,x=0,\;2\pi ,\;X_{S}(x,0)=2.5,\;X_{I}(x,0)=0.1,\;Y(x,0)=0.085,\;x\in (0,\;2\pi ).$$

By calculating, we have

$$\begin{array}{c} M_{1}^{*}\approx 2.5581,\;M_{1}=2.5582,\;M_{2}^{*}\approx 0.2031\;,M_{2}=0.2032,\;M_{3}^{*}\approx 0.1761,\;M_{3}=0.1762,\\ m_{1}^{*}\approx 1.7244,\;\;m_{1}=1.7243,\;\;m_{2}^{*}\approx 0.0301,\;\;m_{2}=0.0300,\;\;m_{3}^{*}\approx 0.0046,\;\;m_{3}=0.0045, \end{array}$$

$$(H_{1})\;\psi^{m}M_{1}-\delta^{l}\approx 2.1032,\;(H_{2})\;\gamma_{1}^{m}M_{2}-r_{2}^{l}\approx 0.6761,$$

$$(H_{3})\;r_{1}^{l}-(b^{m}+\beta^{m})M_{2}\approx 12.5787,\;\;(H_{4})\;\psi^{l}m_{1}-(\alpha^{m}M_{3}+\delta^{m})\approx 3.0363,$$

$$(H_{5})\;\frac{\gamma^{l}m_{2}}{d^{m}(1+a^{m}M_{2}+e^{m}M_{3})}-\frac{r_{2}^{m}}{d^{m}}\approx 0.0046,\;(H_{6})\;b^{l}-\psi^{m}\approx 2.706,$$

$$(H_{7})\;c^{l}-b^{m}-\beta^{m}-\frac{a^{m}\alpha^{m}M_{3}+\gamma^{m}(1+e^{m}M_{3})}{{\left(1+a^{l}m_{2}+e^{l}m_{3}\right)}^{2}}\approx 0.5622,\;(H_{8})\;d^{l}-\frac{\alpha^{m}+a^{m}\alpha^{m}M_{2}-\gamma^{l}e^{l}m_{2}}{{\left(1+a^{l}m_{2}+e^{l}m_{3}\right)}^{2}}\approx 3.3328.$$

It is quite clear that model (18)–(19) satisfy the assumptions of Theorem 6. From Theorem 6 it is easy to know that model (18)–(19) have a strictly positive two-periodic spatially homogeneous periodic solution $(X_{S}^{*}(t),X_{I}^{*}(t),Y^{*}(t)).$ Moreover, the solution $\left(X_{S}(x,t),X_{I}(x,t),Y(x,t)\right)$ of model (17)–(18) fulfills


$$\underset{t\to \infty }{\lim}\left(X_{S}(x,t)-X_{S}^{*}(t)\right)=0,\;\underset{t\to \infty }{\lim}\left(X_{I}(x,t)-X_{I}^{*}(t)\right)=0,\;\underset{t\to \infty }{\lim}\left(Y(x,t)-Y^{*}(t)\right)=0,\;\mathrm{uniformly}\;\mathrm{for}\;x\in (0,2\pi ).$$

**Figure 1:**
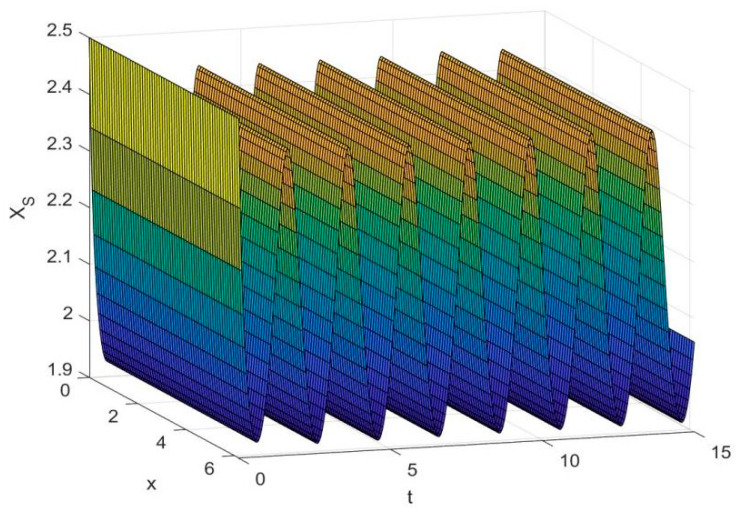
Temporal dynamics of species densities $X_{S}(x,t)$ in model (18)–(19).

**Figure 2:**
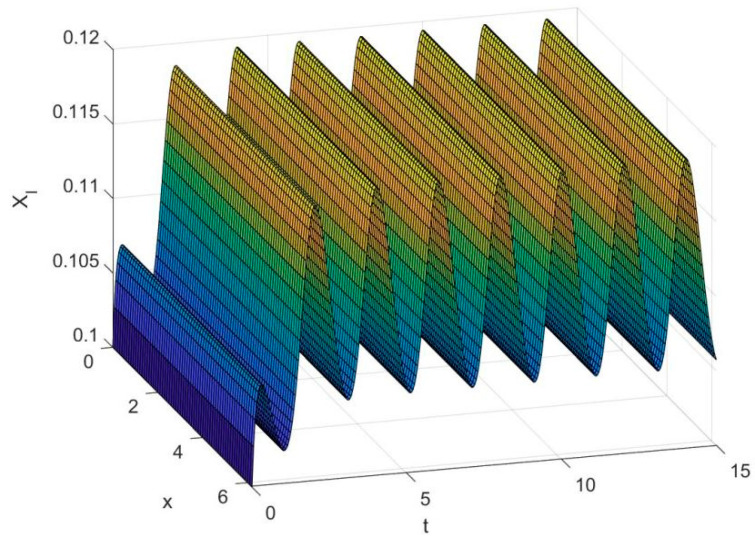
Temporal dynamics of species densities $X_{I}(x,t)$ in model (18)–(19).

**Figure 3:**
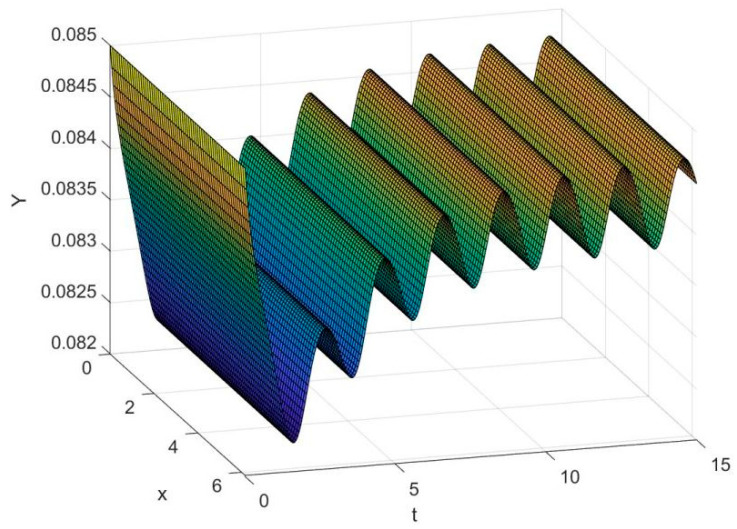
Temporal dynamics of species densities Y(x,t) in model (18)–(19).

**Figure 4:**
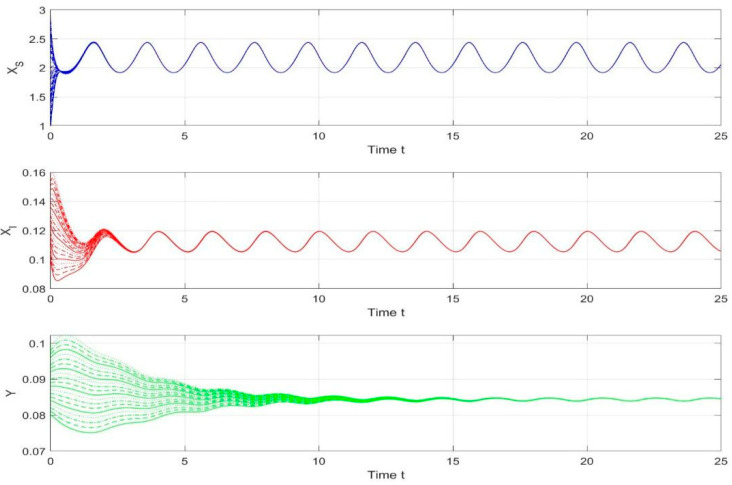
Phase plane trajectories of numerical solutions for model (18)–(19) with fixed spatial variable x=0.6π and different initial values.

**Figure 5:**
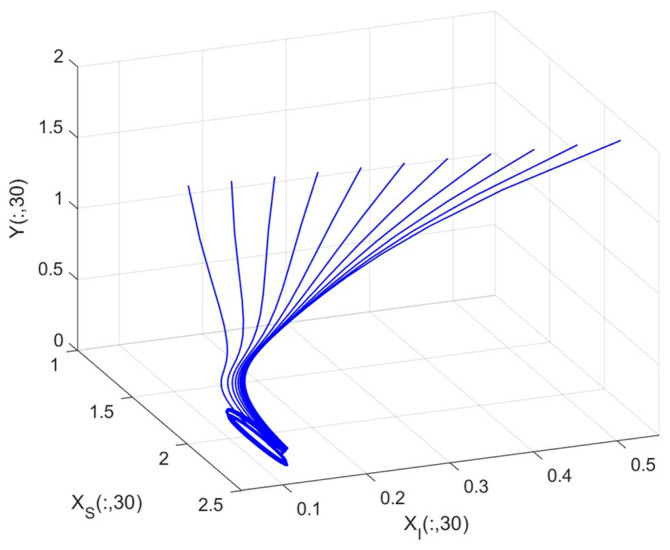
Temporal evolution of population densities $X_{S},X_{I},Y$ in model (18)–(19) under different positive initial values with fixed spatial variable x=0.6π.

**Figure 6:**
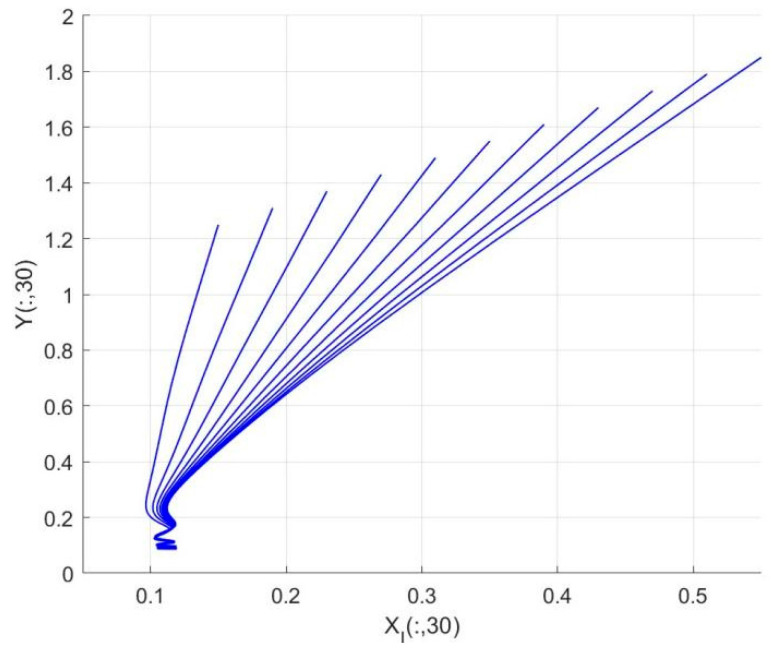
Temporal evolution of population densities $X_{I},Y$ in model (18)–(19) under different positive initial values with fixed spatial variable x=0.6π.

**Figure 7:**
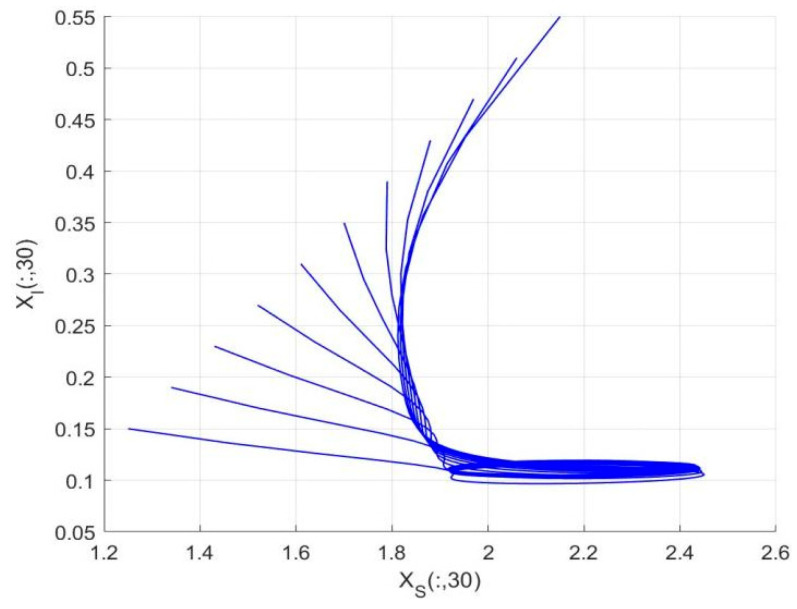
Temporal evolution of population densities $X_{S},X_{I}$ in model (18)–(19) under different positive initial values with fixed spatial variable x=0.6π.

**Figure 8:**
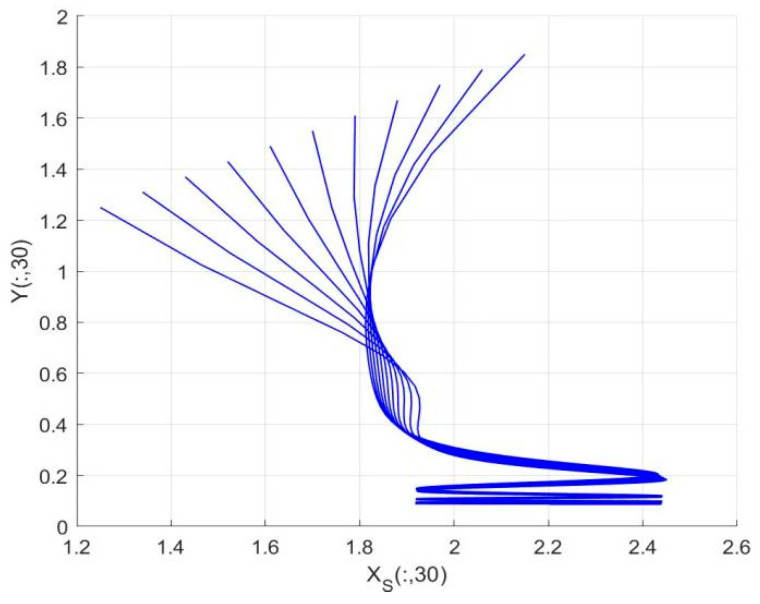
Temporal evolution of population densities $X_{S},Y$ in model (18)–(19) under different positive initial values with fixed spatial variable x=0.6π.

By employing the finite difference method and the MATLAB 7.1 software package, numerical solutions for systems (18)–(19) are obtained and illustrated in Figure 1, Figure 2, Figure 3 and Figure 4. These figures clearly indicate the existence of a strictly positive, globally asymptotically stable, spatially homogeneous periodic solution in model (18)–(19). To further verify the global asymptotic stability of this solution, extensive numerical simulations were conducted using a wide range of positive initial values. The results consistently confirm that the two-periodic solution remains asymptotically stable irrespective of the initial conditions selected. Additional supporting results are provided in Figure 5, Figure 6, Figure 7 and Figure 8.

Both the theoretical results from Theorem 6 and the numerical simulations presented in Figure 1, Figure 2, Figure 3 and Figure 4 indicate that the two-species nonautonomous reaction–diffusion model (2)–(3), which include disease dynamics in the prey population and a Beddington–DeAngelis functional response, can reach an equilibrium under conditions where the intrinsic growth rate and infection rate of the susceptible prey are sufficiently high, and the energy conversion efficiency of the predator is also high. Specifically, in model (2)–(3), the population densities of susceptible prey, infected prey, and predators converge to a spatially homogeneous state and exhibit periodic oscillations with a period of 2 over extended time. Furthermore, as supported by Figure 5, Figure 6, Figure 7 and Figure 8, for any set of positive initial values, as long as the criteria stated in Theorem 6 are met, the solution trajectories of model (2)–(3) consistently approach a stable non-constant positive periodic solution. This study establishes a significant connection between abstract mathematical theories and applied conservation biology. The rigorous population dynamics framework developed herein not only enhances our fundamental understanding of ecological complexity but also offers practical implications for biodiversity preservation under conditions of environmental uncertainty.

Taking into account the natural phenomenon in real ecosystems where a very small number of susceptible prey may also fall prey to predators, we incorporated a weak predation term for the susceptible prey population into the original model and obtained the following model.

(20) $$\begin{array}{cc} \frac{\partial X_{S}(x,t)}{\partial t}-\Delta X_{S}(x,t) & =X_{S}(x,t)[(10+\sin\pi t)-(4.4+0.1\sin\pi t)X_{S}(x,t)-((4.4+0.1\sin\pi t)\\ & \;\;+(1.5+0.1\sin\pi t))X_{I}(x,t)\\ & \;\;-\frac{\left(0.01+0.001\sin\pi t)Y(x,t\right)}{1+(0.007+0.01\sin\pi t)X_{S}(x,t)+(0.04+0.01\sin\pi t)Y(x,t)}],\\ \frac{\partial X_{I}(x,t)}{\partial t}-\Delta X_{I}(x,t) & =X_{I}(x,t)[-(2+0.01\sin\pi t)-(10.45+0.1\sin\pi t)X_{I}(x,t)\\ & \;\;+(1.5+0.1\sin\pi t)X_{S}(x,t)\\ & \;\;-\frac{\left(0.5+0.01\sin\pi t)Y(x,t\right)}{1+(0.007+0.01\sin\pi t)X_{I}(x,t)+(0.04+0.01\sin\pi t)Y(x,t)}],\\ \frac{\partial Y(x,t)}{\partial t}-\Delta Y(x,t) & =Y(x,t)[-(0.08+0.01\sin\pi t)-(3.85+0.01\sin\pi t)Y(x,t)\\ & \;\;+\frac{\left(3.65+0.02\sin\pi t)X_{I}(x,t\right)}{1+(0.007+0.01\sin\pi t)X_{I}(x,t)+(0.04+0.01\sin\pi t)Y(x,t)}\\ & \;\;+\frac{\left(2.65+0.02\sin\pi t)X_{S}(x,t\right)}{1+(0.007+0.01\sin\pi t)X_{S}(x,t)+(0.04+0.01\sin\pi t)Y(x,t)}]. \end{array}$$

**Figure 9:**
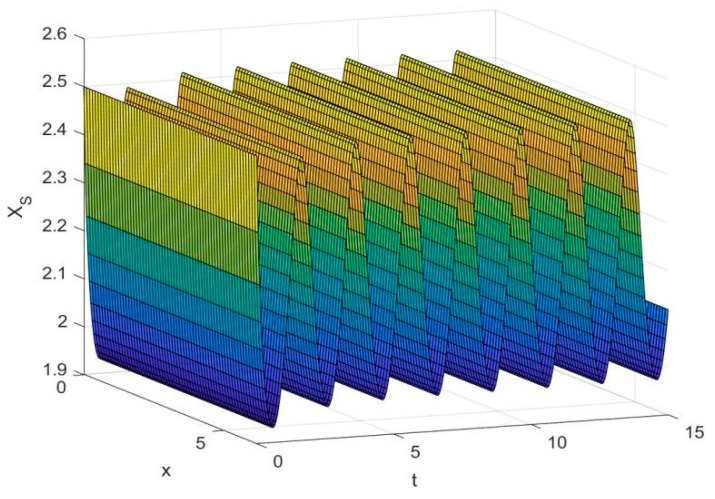
Temporal dynamics of species densities $X_{S}(x,t)$ in model (19)–(20).

**Figure 10:**
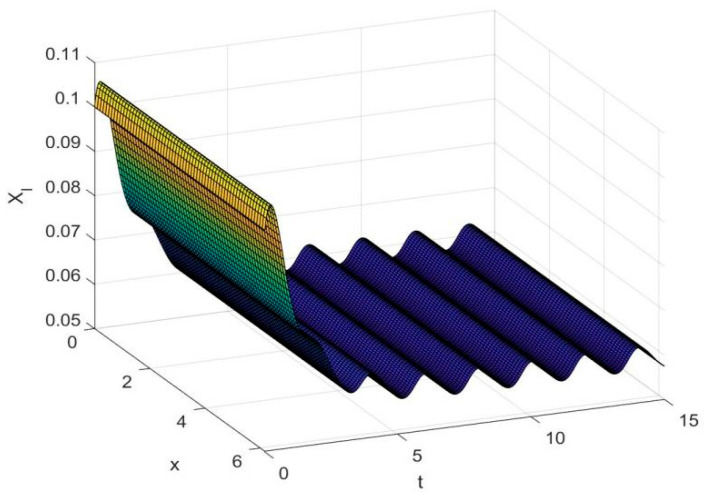
Temporal dynamics of species densities $X_{I}(x,t)$ in model (19)–(20).

**Figure 11:**
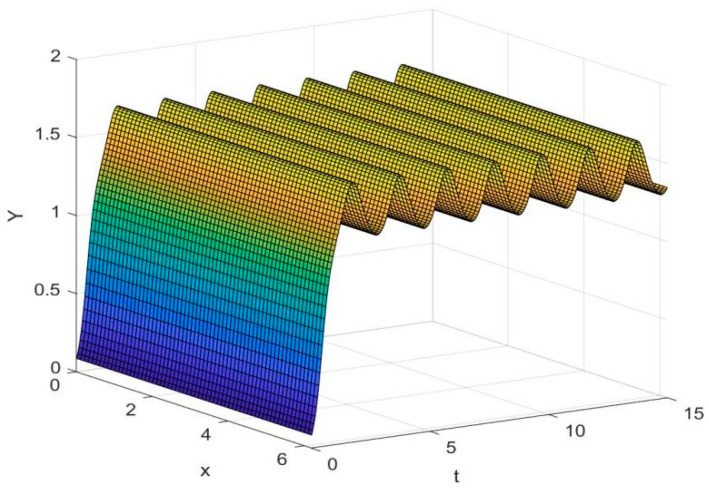
Temporal dynamics of species densities Y(x,t) in model (19)–(20).

**Figure 12:**
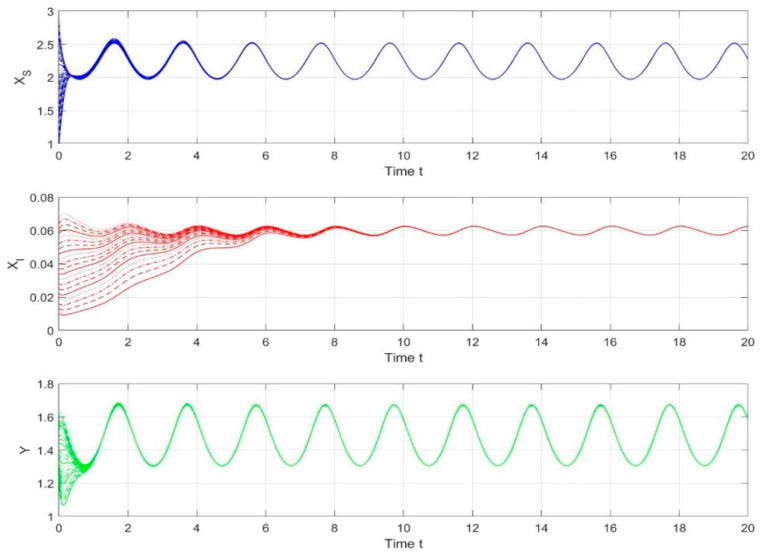
Phase plane trajectories of numerical solutions for model (19)–(20) with fixed spatial variable x=0.6π and different initial values.

**Figure 13:**
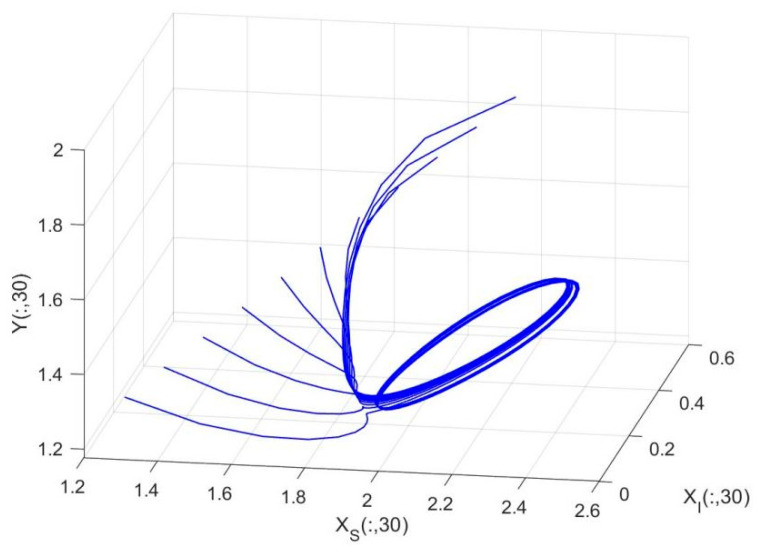
Temporal evolution of population densities $X_{S},X_{I},Y$ in model (19)–(20) under different positive initial values with fixed spatial variable x=0.6π.

**Figure 14:**
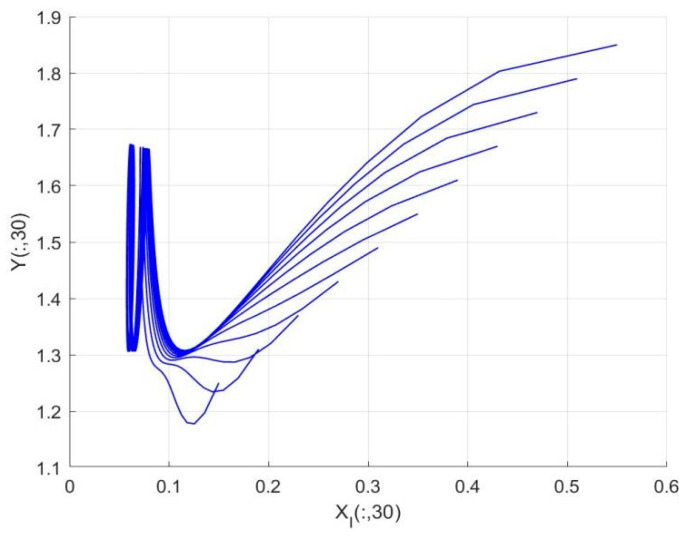
Temporal evolution of population densities $X_{I},Y$ in model (19)–(20) under different positive initial values with fixed spatial variable x=0.6π.

**Figure 15:**
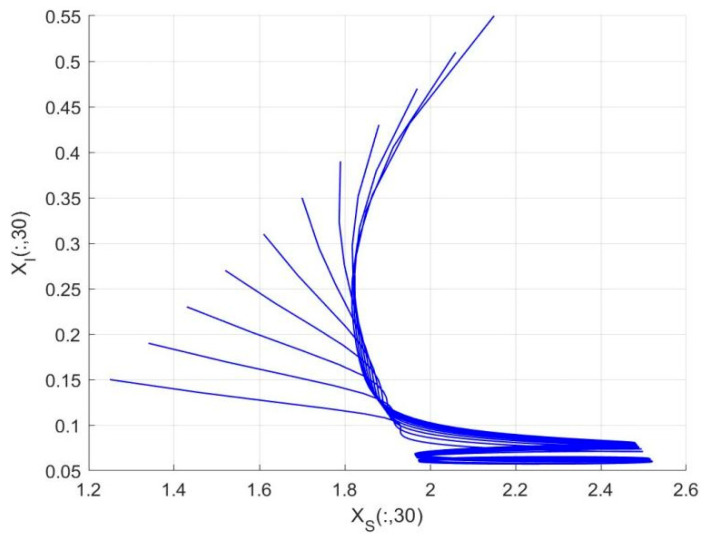
Temporal evolution of population densities $X_{S},X_{I}$ in model (19)–(20) under different positive initial values with fixed spatial variable x=0.6π.

**Figure 16:**
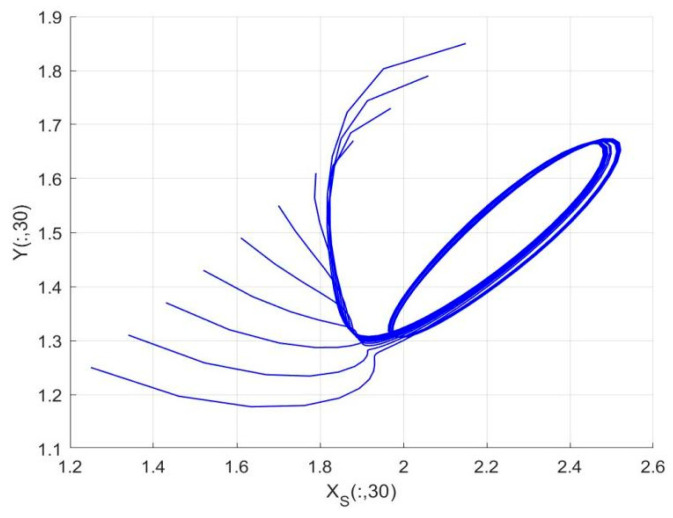
Temporal evolution of population densities $X_{S},Y$ in model (19)–(20) under different positive initial values with fixed spatial variable x=0.6π.

By employing the finite difference method and the MATLAB 7.1 software package, numerical solutions for systems (19)–(20) are obtained and illustrated in Figure 9, Figure 10, Figure 11, Figure 12, Figure 13, Figure 14, Figure 15 and Figure 16. By comparing Figure 1, Figure 2, Figure 3, Figure 4, Figure 5, Figure 6, Figure 7 and Figure 8 with Figure 9, Figure 10, Figure 11, Figure 12, Figure 13, Figure 14, Figure 15 and Figure 16, we found that after introducing the weak predation term for susceptible prey into the original model, the predator population increased significantly, while the number of infected prey decreased markedly. Meanwhile, the number of susceptible prey showed little change, yet the persistence and stability of the ecosystem remained unaltered. Since the number of susceptible prey far exceeds that of prey infected with the disease, these numerical solutions are in line with actual ecological natural phenomena. The above numerical simulation results further illustrate the complexity of the dynamic properties of ecosystems and also demonstrate that our research question holds significant theoretical and practical implications.

## 6. Discussion

This study marks a substantial advancement in eco-epidemiological modeling by seamlessly integrating disease dynamics, spatial dispersal, and predator–prey interactions within a Beddington–DeAngelis functional response framework. The theoretical achievements, encompassing the proof of global solution existence, the formulation of species persistence criteria, and the analysis of asymptotic stability in nonautonomous periodic environments, collectively establish a rigorous foundation for deciphering the complexities of multi-species ecological systems. From a methodological perspective, the development of innovative Lyapunov functions, specifically designed to address the challenges posed by time-varying dynamics and epidemic characteristics, fills a crucial void in the stability analysis of eco-epidemiological models. These functions provide robust and reliable tools for conducting long-term ecological forecasts. Furthermore, numerical simulations corroborate the model’s convergence and stability within defined density boundaries, thereby enhancing its credibility and practical applicability.

(1) Practical Implications and Policy Relevance

Beyond theoretical contributions, this study offers actionable insights for addressing urgent ecological and public health challenges. First, the identified critical thresholds for ecosystem resilience empower managers to implement proactive interventions, such as habitat restoration or population controls, to avert collapse—a priority for preserving biodiversity in the face of anthropogenic pressures. Second, the analytical framework for disease persistence in spatially structured environments enables wildlife managers to predict transmission pathways across habitats and prey species. This capability is invaluable for designing targeted interventions, such as vaccination programs or movement restrictions, that minimize unintended ecological harm. Third, the stability criteria provide evidence-based guidance for resource allocation during disease outbreaks, ensuring prioritization of high-impact control measures. By highlighting keystone species, the model also aids in maintaining ecological balance, which is critical for ecosystem service provision.

A representative numerical example underscores these practical benefits. By simulating population trajectories under varying initial conditions, the model reveals species at risk of decline, enabling managers to prioritize conservation efforts. For instance, inputting regional wildlife data could identify habitats requiring urgent protection or disease surveillance. Such applications align with policy needs for sustainable ecosystem management, offering a scientific basis for balancing conservation with human development. For policymakers, the model provides a quantitative tool to craft legislation that protects biodiversity while accounting for economic and social factors, fostering harmonious coexistence between human activities and natural systems.

(2) Limitations and Future Directions

Despite its advancements, this study has limitations that open avenues for future research. The current framework assumes homogeneous pathogen effects within prey populations, but real-world systems often involve pathogens infecting both predators and prey or multi-trophic interactions. Expanding the model to include cross-species infections or three-tier trophic systems (e.g., predator–prey–plant dynamics) would enhance its ecological realism. Additionally, while the Beddington–DeAngelis response effectively captures intermediate predation complexity, exploring alternative functional responses, including ratio-dependent or Holling type III mechanisms, has the potential to uncover subtle predation dynamics that emerge under environmental changes like climate change or habitat fragmentation.

Another critical area is the interplay between time delays (e.g., disease incubation periods) and spatial diffusion. Time delays may induce oscillatory dynamics or destabilize equilibria, while spatial heterogeneity can alter disease spread patterns. Investigating these combined effects would deepen our understanding of long-term ecological equilibria. Finally, empirical validation using real-world datasets remains essential. Field studies tracking predator–prey populations and disease prevalence across landscapes could refine the model parameters and test predictive accuracy, strengthening its utility for conservation strategies.

## 7. Conclusions

This research has achieved a significant breakthrough in eco-epidemiological modeling by introducing a comprehensive framework that integrates disease dynamics, spatial dispersal, and predator–prey interactions using the Beddington–DeAngelis functional response as its theoretical foundation. Through systematic theoretical analysis, we have established a solid basis for understanding complex multi-species interactions within ecosystems. This includes proving the existence of global solutions, establishing criteria for species persistence, and demonstrating asymptotic stability in dynamic nonautonomous periodic environments. The methodological advances in this study, particularly the development of custom Lyapunov functions, have substantially improved our ability to analyze model stability under time-varying and epidemic-prone conditions. These analytical tools not only deepen theoretical understanding but also enable reliable long-term ecological forecasting, effectively connecting theoretical concepts with practical applications. The practical implications of these findings are extensive, providing valuable insights for both ecosystem management and public health policy. By identifying critical thresholds for ecosystem resilience, our model supplies managers with scientific evidence to implement preventive measures against ecological collapse. This is particularly crucial given increasing anthropogenic pressures. Additionally, the analytical framework for disease persistence in spatially structured environments allows wildlife managers to anticipate and reduce disease transmission across habitats and species, thereby minimizing unintended ecological consequences.

Looking ahead, while this study has established a solid foundation, it also identifies promising directions for future investigation. The assumption of homogeneous pathogen effects within prey populations, though mathematically convenient, may not fully reflect the complexity of natural ecosystems. Subsequent research could explore cross-species transmission patterns and multi-trophic interactions to enhance the model’s ecological realism. Furthermore, the impacts of environmental changes including climate change and habitat fragmentation on predation dynamics deserve deeper examination, potentially through developing alternative functional responses. The relationship between temporal factors such as disease incubation periods and spatial diffusion mechanisms constitutes another valuable research direction. Understanding how these elements collectively shape ecological equilibria will advance our knowledge of ecosystem stability and resilience. Empirical validation through field studies remains crucial for parameter refinement and predictive accuracy assessment, ultimately strengthening the model’s practical utility in conservation planning and policy formulation. This study thus represents a pivotal advancement in eco-epidemiological modeling, delivering both theoretical innovations and analytical tools to address pressing ecological and public health challenges. As this research trajectory evolves, it promises to support evidence-based ecosystem management and policy decisions, fostering a more sustainable coexistence between human societies and natural systems.

## Figures and Tables

**Table 1:** The biological meaning associated with the parameters in model (1).

Parameter	Biological Meaning
$r_{1}(t)$	Intrinsic growth rate susceptible prey populations
b(t)	Measure of the strength of prey population interference competition
β(t)	Transmission coefficient from susceptible prey to infected prey
δ(t)	Death rate of infected prey including natural death rate and disease related death rate in the absence of predator
c*(t)	Measure of the strength of infected prey population interference competition arises from their scramble for shelters such as caves. In contrast, the healthy prey population, which moves swiftly and can thus evade predators, does not engage in competition for such shelters. Predators exclusively prey upon infected individuals and do not consume susceptible prey, please see assumption (viii)
ε(t)	Measure of the strength of infected prey population interference competition based on food. According to assumptions (ii) and (iv), the disease that infected preys contract is an incurable and fatal one, and infected preys die before reaching adulthood, meaning they have a relatively short survival time. As a result, the intraspecific competition among infected preys due to food is very weak. That is to say, the parameter ε(t) $\mathrm{is}\;\mathrm{much}\;\mathrm{smaller}\;\mathrm{than}\;\mathrm{parameters}\;c^{*}(t)$ and β(t)
α(t)	Maximum value of per-capita reduction rate of infected prey population due to predation
a(t)	Time for the prey to be digested and attracted
e(t)	Measure of the strength of interference between predators during hunting
γ(t)	Energy conversion rate of prey after predation
$r_{2}(t)$	Natural death rate of predator
d(t)	Measure of the strength of predator population interference competition

## Data Availability

Data are contained within the article.
